# The Power of Gene-Based Rare Variant Methods to Detect Disease-Associated Variation and Test Hypotheses About Complex Disease

**DOI:** 10.1371/journal.pgen.1005165

**Published:** 2015-04-23

**Authors:** Loukas Moutsianas, Vineeta Agarwala, Christian Fuchsberger, Jason Flannick, Manuel A. Rivas, Kyle J. Gaulton, Patrick K. Albers, GoT2D Consortium, Gil McVean, Michael Boehnke, David Altshuler, Mark I. McCarthy

**Affiliations:** 1 Wellcome Trust Centre for Human Genetics, University of Oxford, Oxford, United Kingdom; 2 Program in Biophysics, Harvard University, Cambridge, Massachusetts, United States of America; 3 Program in Medical and Population Genetics, Broad Institute of Harvard and MIT, Cambridge, Massachusetts, United States of America; 4 Department of Biostatistics and Center for Statistical Genetics, University of Michigan, Ann Arbor, Michigan, United States of America; 5 Center for Human Genetic Research, Massachusetts General Hospital, Boston, Massachusetts, United States of America; 6 Department of Genetics, Harvard Medical School, Boston, Massachusetts, United States of America; 7 Department of Biology, Massachusetts Institute of Technology, Cambridge, Massachusetts, United States of America; 8 Oxford Centre for Diabetes, Endocrinology and Metabolism, University of Oxford, Oxford, United Kingdom; Institute for Molecular Medicine Finland, FIMM, University of Helsinki, FINLAND

## Abstract

Genome and exome sequencing in large cohorts enables characterization of the role of rare variation in complex diseases. Success in this endeavor, however, requires investigators to test a diverse array of genetic hypotheses which differ in the number, frequency and effect sizes of underlying causal variants. In this study, we evaluated the power of gene-based association methods to interrogate such hypotheses, and examined the implications for study design. We developed a flexible simulation approach, using 1000 Genomes data, to (a) generate sequence variation at human genes in up to 10K case-control samples, and (b) quantify the statistical power of a panel of widely used gene-based association tests under a variety of allelic architectures, locus effect sizes, and significance thresholds. For loci explaining ~1% of phenotypic variance underlying a common dichotomous trait, we find that all methods have low absolute power to achieve exome-wide significance (~5-20% power at α=2.5×10^-6^) in 3K individuals; even in 10K samples, power is modest (~60%). The combined application of multiple methods increases sensitivity, but does so at the expense of a higher false positive rate. MiST, SKAT-O, and KBAC have the highest individual mean power across simulated datasets, but we observe wide architecture-dependent variability in the individual loci detected by each test, suggesting that inferences about disease architecture from analysis of sequencing studies can differ depending on which methods are used. Our results imply that tens of thousands of individuals, extensive functional annotation, or highly targeted hypothesis testing will be required to confidently detect or exclude rare variant signals at complex disease loci.

## Author Summary

Re-sequencing technologies allow for a more complete interrogation of the role of human variation in complex disease. The inadequate power of single variant methods to assess the role of less common variation has led to the development of numerous statistical methods for testing aggregate groups of variants for association with disease. Such endeavors pose substantial analytical challenges, however, due to the diverse array of genetic hypotheses that need to be considered. In this work, we systematically quantify and compare the performance of a panel of commonly used gene-based association methods under a range of allelic architectures, significance thresholds, locus effect sizes, sample sizes, and filters for neutral variation. We find that MiST, SKAT-O, and KBAC have the highest mean power across simulated datasets. Across all methods, however, the power to detect even loci of relatively large effect is very low at exome-wide significance thresholds for sample sizes comparable with those of ongoing sequencing studies; as such, the absence of signal in studies of a few thousand individuals does not exclude a role for rare variation in complex traits. Finally, we directly compare the results reported by different gene-based methods in order to identify their comparative advantages and disadvantages under distinct locus architectures. Our findings have implications for meaningful interpretation of both positive and negative findings in ongoing and future sequencing studies.

## Introduction

To assess whether a single variant at a locus contributes to disease risk, the statistical analysis framework is relatively straightforward: compare the frequencies of alleles or genotypes at the site in relation to phenotype. To assess whether multiple variants in the same gene contribute to disease, a much larger array of potential genetic models must be considered. If the causal alleles are rare (defined here as MAF<1%), then power to detect each variant’s effect individually is limited. For example, power to detect a variant with MAF = 0.5% and relative risk (RR) = 3 in 3K case-control samples (1.5K cases and 1.5K controls) at α = 5×10^-8^ is ~5% [1]. Variants that are private to individuals, as some deleterious mutations are hypothesized to be, present greater challenges yet. As a result, numerous statistical methods have been developed in recent years to test aggregate groups of rare variants for association to disease [2–4].

Re-sequencing experiments have identified a handful of rare variants which modulate risk for common, complex diseases. Examples include variants in *NOD2* for Crohn’s disease (4 variants with MAF 0.1–0.8%, ORs 1.4–4.0, detected by single variant association)[5], *PCSK9* for coronary heart disease (2 variants with MAF 0.8 and 1.8%, OR ~0.1, detected by single variant association)[6], *LPL* for hypertriglyceridemia (154 missense variants with MAF<1%, present in cases, detected using the T1 gene-based association method)[7], and *MTNR1B* for type 2 diabetes (13 functionally-screened variants with MAF<0.1%, collective OR ~5.5, detected using the KBAC gene-based method)[8]. Each of these disease loci is characterized by different numbers, frequencies, and effect sizes of rare variants, but in each of these examples, the estimated proportion of phenotypic variance explained per locus is ~0.5–1.5%.

As large-scale (e.g. genome-wide or exome-wide) studies are now being conducted in hundreds and thousands of individuals, several questions emerge. If loci similar to *LPL* or *MTNR1B* exist undiscovered across the genome, what is the power of different gene-based methods to detect them? What effect sizes are studies of a given sample size well-powered to detect? To what extent does power depend on the underlying architecture of causal allelic variation, and how should researchers navigate through the ensemble of available gene-based tests? To interpret the results of gene-based association methods in sequencing studies, it is critical to quantify the power of each method to detect signals under a range of hypothesized locus architectures.

Although the introduction of each novel gene-based association test has typically been accompanied by evaluation of the test’s performance alongside alternatives, each such analysis has compared different subsets of tests, made different assumptions about locus architecture and study design, and employed different simulation approaches. Comparative studies on the relative power of different methods [9–11], while informative, have used small sample sizes, simulated limited locus architectures (e.g., with fixed numbers of causal variants) that may not be representative of complex diseases, and considered only nominal levels of significance (α>0.01). Thus, further work is required to determine how different gene-based tests perform under different genetic models of complex disease.

In this study, we systematically explore the power of eleven currently available and widely-used gene-based association methods to detect rare variant signals drawn from a range of principled genetic architectures of disease, in sample sizes consistent with those of ongoing re-sequencing studies. We assess the impact of locus architecture, effect size, and functional variant filters on the power of each method at stringent levels of significance. By evaluating all tests together at loci simulated under a range of continuous frequency-effect size distributions, we characterize each method’s success and failure modes, and describe genetic hypotheses for which particular methods may be better powered than others.

## Results

We first developed a simulation approach to evaluate the power of each gene-based method. We assumed two key requirements for simulations to be informative: 1) simulated genetic variation must approximate the site frequency spectrum (SFS) and haplotype structure of empirical data, and 2) the distribution of relative risks by frequency class should correspond to hypotheses about the genetic architecture of disease that are compatible with observation.

**Fig 1:**
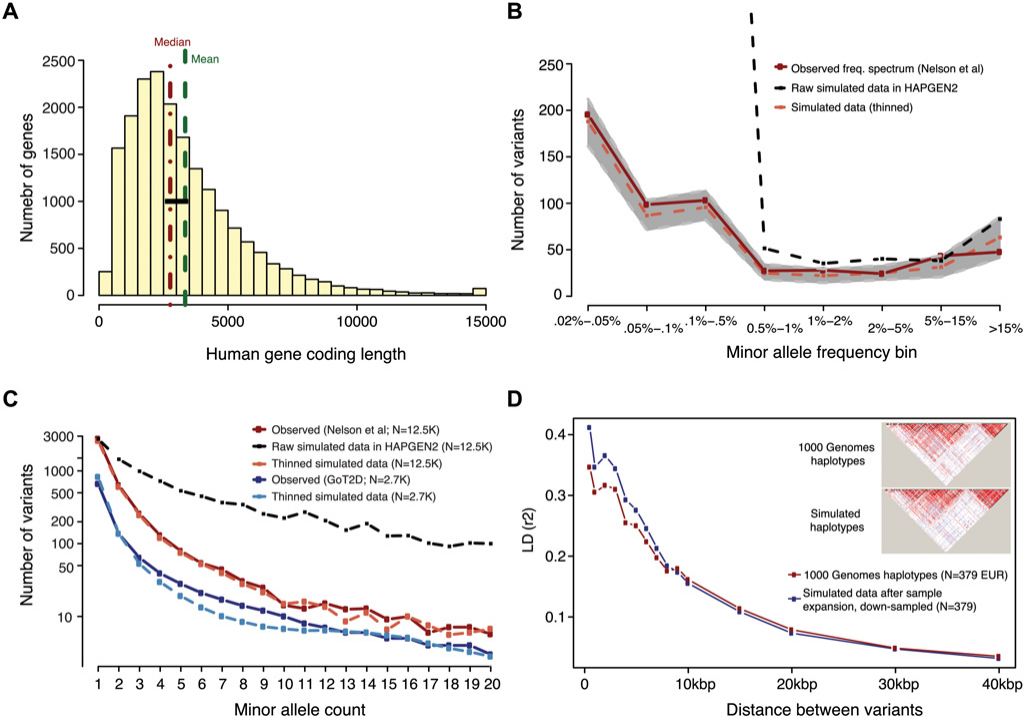
Generation of simulated genotype data at human gene loci in large sample sizes with HAPGEN2. Haplotypes were simulated at ‘average’ human protein-coding genes drawn from the center of the distribution of RefSeq gene total exon length **(A)**. Vertical dotted lines in red and green indicate the median and mean values of exon length, respectively. Black bar represents the 24 genes selected for simulation. **(B,C)** Site frequency spectrum of simulated data, as compared to observed human data. Data were simulated via staged expansion of 1000 Genomes Project haplotypes using the HAPGEN2 software; the mutation parameter was fit to match the site frequency spectrum of protein-coding variation observed in exome sequencing studies, e.g. as reported Nelson et al 2012. Raw simulated data from HAPGEN2 in large sample sizes produced an excess of rare sites; these were down-sampled to match observed data. The grey area in **(B)** represents the [5%, 95%] interval across all simulated genes, obtained using bootstrapping. The site frequency spectrum of simulated data in a smaller sample size (N = 2.7K) also matched an independent set of observed exome sequencing data from the GoT2D consortium **(C)**. Haplotype structure, as measured by linkage disequilibrium between variants, was also preserved in the simulated data after sample expansion **(D)**. The inset shows a representative example of simulations at the GATA3 gene locus.

To achieve these objectives, we employed the program HAPGEN2 [12] to simulate variation across the full SFS in thousands of individuals and build a phased reference panel with more individuals than are publicly available at present for a single ethnic group. We started with phased haplotypes from 379 European individuals (1000G Project Phase 1, release 3) [13]. To expand this reference panel to a larger number of individuals, we applied a staged, iterative approach which preserves linkage disequilibrium structure between relatively common variants while introducing new low-frequency variants upon the original haplotypes to match the empirical SFS observed at exonic regions of 202 genes in a study of >12K individuals of European ancestry [14] (S1 Text and Figs 1B, 1C, 1D and S1 and S2). All simulations were performed on 24 human genes of average coding length on chromosome 10 (Fig 1A and S1 Table). While gene coding length does likely contribute to the power to detect association signals, the selection of genes with average length in this study enabled us to conduct controlled characterization of the effects of locus architecture on power.

We modeled the complex disease type 2 diabetes (T2D, assuming prevalence 8%), and introduced phenotypic effects (relative risk per variant, assuming additive effects) by sampling up to 35 exonic causal variants per locus (variant cap imposed due to software limitations, see Methods) from six different joint distributions of causal variant frequencies and effect sizes (S3 and S4 Figs and Tables 1 and S2). These distributions were obtained from forward simulations of global genetic architecture under different disease models that are consistent with properties of empirical sequence variation and the observed prevalence and heritability of T2D [15]. The three main architectures assume strong (AR1), moderate (AR2), or weak (AR3) purifying selection against causal alleles. Broadly, AR1 results in a sharp inverse correlation between variant frequency and effect size, AR2 produces modest correlation, and AR3 is characterized by rare and common alleles that have similar additive effects on phenotype. AR4 and AR5 are variations of AR1 and AR2, respectively, in which only rare (MAF<1%) variants at a locus contribute to disease. AR6 assumes a frequency-effect size map identical to AR2, but assigns a 50%-50% mixture of risk and protective effects; this represents the hypothesis that some variants in a gene increase disease risk, while other variants in the same gene have protective effects.

**Table 1:** Locus architectures modeled at simulated loci.

Simulated architecture	Direction of effects	Causal variant frequencies	Selection on causal alleles
**AR1**	All deleterious	Across full SFS	Strong
**AR2**	All deleterious	Across full SFS	Moderate
**AR3**	All deleterious	Across full SFS	Weak
**AR4**	All deleterious	MAF <1%	Strong
**AR5**	All deleterious	MAF <1%	Moderate
**AR6**	50% deleterious, 50% protective	Across full SFS	Moderate

We evaluated a set of eleven gene-based association methods (CMC [16], VT [17], FRQWGT [18], WILCOX-WSS [19], KBAC [20], BURDEN [18], UNIQ [18], C-ALPHA [21], SKAT [22], SKAT-O [23], and MiST [24]; see Table 2) on these simulated datasets. The tests we applied can be broadly categorized as unidirectional ‘burden’ tests, bidirectional variance-component tests (SKAT, C-ALPHA), and linear combinations of these two classes (SKAT-O, MiST). The unidirectional tests can be further sub-divided into collapsing regression methods (CMC), weighted sum methods (FRQWGT, KBAC, WILCOX-WSS, VT), and permutation-based summary count methods (BURDEN, UNIQ). We selected this set of tests because they represent a broad range of analytical approaches, most of which are readily available in the widely-used software packages PLINK/Seq [18] and EPACTS [25]. Before further evaluation, we confirmed that all tests were well-calibrated, at α = 0.05 and 10^-4^, in (null) datasets where no variants were assigned any causal effects (S5 Fig).

**Table 2:** Published gene-based rare variant association methods evaluated.

Method name	Citation	Software	Description
***Unidirectional rare variant gene-based tests***			
*Collapsing methods*			
Combined Multivariate and Collapsing (CMC)	Liu & Leal, PLoS Comp. Bio. 2008	EPACTS	All rare variants collapsed into a single variant; individual dosage for the collapsed ‘variant’ is regressed against phenotype.
*Weighted and un-weighted sum methods*			
Variable threshold (VT)	Price et al, AJHG. 2010	PLINK-Seq	Sum of rare allele count in cases vs. controls; allele frequency threshold for inclusion is varied to maximize test statistic.
Weighted Sum Statistic (FRQWGT)	Madsen & Browning, PLoS Gen. 2009	PLINK-Seq	Permutation-based test comparing inverse-frequency-weighted rare variant counts per individual in cases vs. controls.
Weighted Sum Method (WILCOX-WSS)	Madsen & Browning, PLoS Gen. 2009	EPACTS	Wilcoxon Rank Sum test between phenotypes and inverse frequency-weighted rare variant scores.
Kernel-Based Adaptive Cluster (KBAC)	Liu & Leal, PLoS Gen. 2010	PLINK-Seq	Variant weights are determined adaptively, and are based on observed effect sizes; individuals scored by weighted sum of allele counts.
*Summary case*:*control count methods*			
BURDEN method	Purcell (PLINK-Seq)	PLINK-Seq	Permutation-based test comparing raw allele counts in cases vs. controls.
UNIQ test	Purcell (PLINK-Seq)	PLINK-Seq	Simple count of total case-unique rare alleles; permutations to assess significance.
***Bi-directional variance-component gene-based tests***			
C-ALPHA	Neale et al, PLoS Gen. 2011	PLINK-Seq	Detects deviation of observed case:control variant counts from expected binomial distribution.
Sequence Kernel Association Test (SKAT)	Wu et al, AJHG 2011	EPACTS	Generalized form of C-ALPHA with variants weighted by allele frequency.
***Linear combination of unidirectional and variance-component tests***			
SKAT-O (‘Optimal’ SKAT)	Lee et al, AJHG. 2012	EPACTS	Adaptive linear combination of unidirectional burden test and variance-component SKAT test.
Mixed Effects Score Test (MiST)	Sun et al, Genetic Epi. 2013	Public R package	Hierarchical regression model combining two independent test statistics which quantify variant effect sizes and ‘heterogeneity’.

### Relative and absolute power of gene-based association methods under different locus architectures

**Fig 2:**
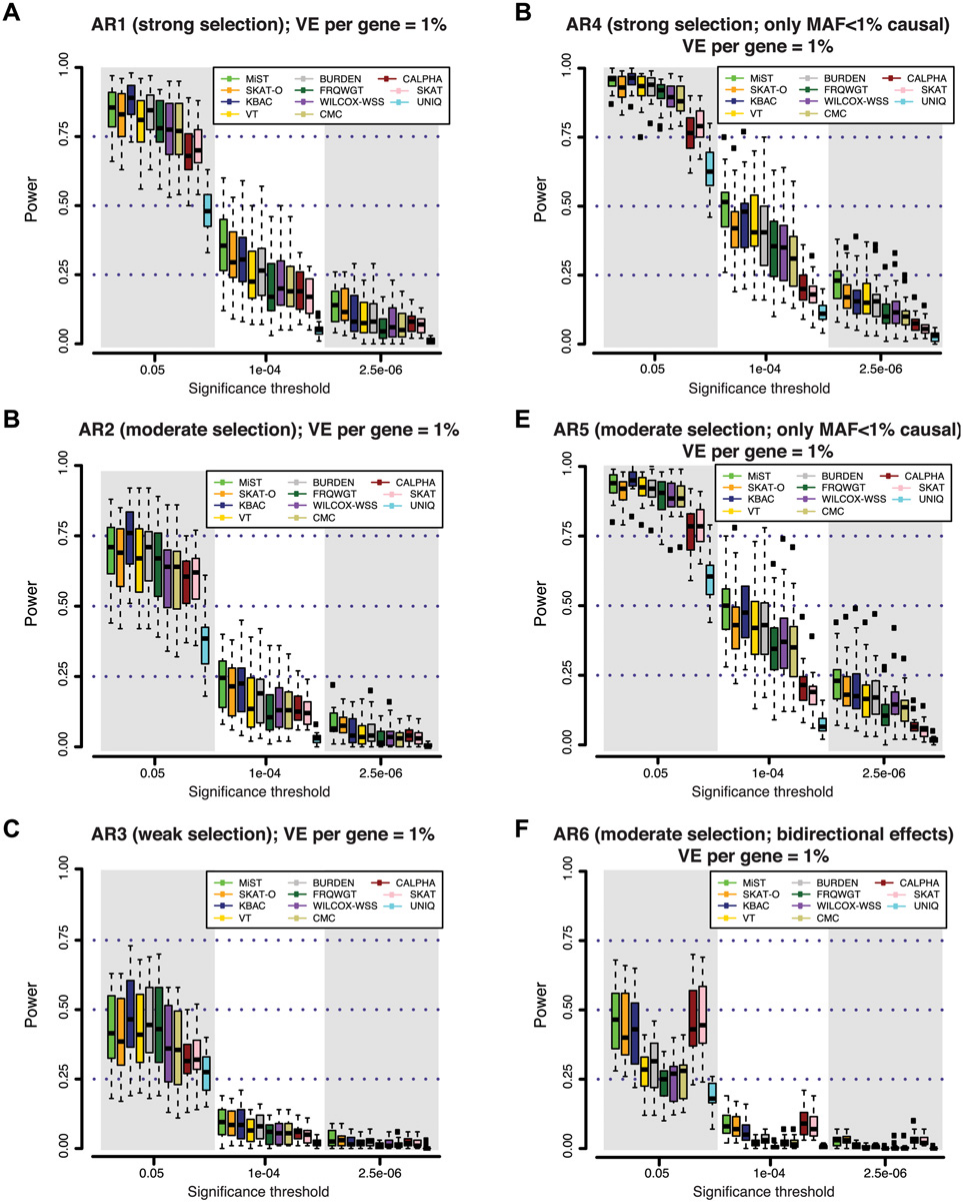
Power of different gene-based rare variant association methods at simulated disease loci. At each gene locus, one hundred independent simulations of phenotypic effects were generated in a sample size of 3K individuals (1.5K cases / 1.5K controls). Variant effects were drawn from varied models of genetic architecture (**A-F**), hypothesizing different degrees of purifying selection against disease alleles (see Methods). Under models with strong selection, there is a strong inverse correlation between variant frequency and effect size; under weak selection rare variant effects are less skewed. At all loci, genetic variants together contribute 1% of the phenotypic variance underlying a trait with common prevalence (8%; modeled as type 2 diabetes). Power is measured as the fraction out of 100 simulations of each gene in which a gene-based test reported a p-value lower than the significance threshold. In (**A-C**), causal variants span the full frequency spectrum (including common alleles), and thus rare alleles account for only a fraction of the locus heritability; in (**D-E**), all causal variants are rare (MAF<1%). In (**F**), causal variants have bi-directional effects (some increase risk of disease, while others reduce risk).

A key question for re-sequencing studies is: what is the power of gene-based association methods to detect causal loci at stringent levels of significance? To address this, we ran each gene-based test at simulated loci explaining 1% of the variance in T2D liability [26, 27] (see Methods) in 1500 cases and 1500 controls (sample size comparable to several recent or ongoing complex trait sequencing studies [28, 29]). Each gene-based test was run on all exonic variants (causal and non-causal) with MAF<1%, unless otherwise stated. The power of each test is shown as a function of significance threshold (α) and architecture in Figs 2, S6, and S7.

In the context of an exome-wide sequencing study, where an appropriate threshold may be α = 2.5×10^-6^ (α = 0.05, after Bonferroni correction for ~20K genes), we found that power is very low (<20%) across all architectures and tests considered. At a less stringent threshold of α = 10^-4^, which might be used to nominate loci for follow-up (under the null, only ~2 such genes would be expected exome-wide), power of the best performing tests across AR1-AR5 remained low (10–50%). This was true irrespective of the allele frequency threshold used for variant inclusion; results for a MAF threshold of 0.5% and 5% are shown in S8 Fig.

We noted that at a nominal level of significance (α = 0.05), many methods had high power (~75%-95%) to detect loci at which deleterious variants (AR1-AR5) explain ~1% of phenotypic variance (Figs 2 and S7). KBAC was consistently the most sensitive method to detect deleterious effects at less stringent levels of significance (up to 95% power at α = 0.05, under AR4). This high sensitivity could be useful in identifying putative signals when only a small number of hypotheses are being tested (e.g. sequencing across only a few targeted loci), or to exclude rare variant models at candidate loci.

Next, we asked whether any of the gene-based methods appear to be uniformly more powerful than others, across the various locus architectures we considered. Under simulated architectures where causal variants all have unidirectional (deleterious) effects (Fig 2A, 2B, 2C, 2D, and 2E **)**, we found that MiST, SKAT-O, and KBAC consistently achieve highest power, while UNIQ is least-powered. However, we did observe differential behavior of these tests depending on the significance threshold: MiST and SKAT-O retained greater power than unidirectional alternatives at stringent thresholds (α<10^-5^), while at less conservative thresholds (α>10^-3^), KBAC was more sensitive (Figs 2A, 2B, 2C, 2D, 2E, 2F and S7).

We next sought to understand how power is influenced by locus architecture. Unsurprisingly, we found that power is higher when the majority of the locus’ total phenotypic effect is due to rare variants included in the association test (e.g. those with MAF<1%). This is evidenced by the gain in power under models with a greater contribution of rare variants: the power of MiST, for example, increased from AR3 (10% at α = 10^-4^ in 3K individuals) to AR2 (23%) to AR1 (36%). Power was higher still under architectures where variants with MAF<1% (i.e. those variants tested) contributed *all* of the locus’ effect (AR4 and AR5): here, the power of MiST was ~50% at α = 10^-4^. Power also depends on the direction of causal effects at a locus: under AR6 (where both risk and protective effects are present), the variance-component tests (SKAT and C-ALPHA) and combined tests (MiST and SKAT-O) were least affected (by design) [21–24] and outperformed all the other methods, retaining ~10% power at α = 10^-4^, while that of unidirectional tests was reduced to <5% (Figs 2F and S7). Finally, we find that power is inversely related to the degree of linkage disequilibrium between causal variants at a locus (S9 Fig).

**Fig 3:**
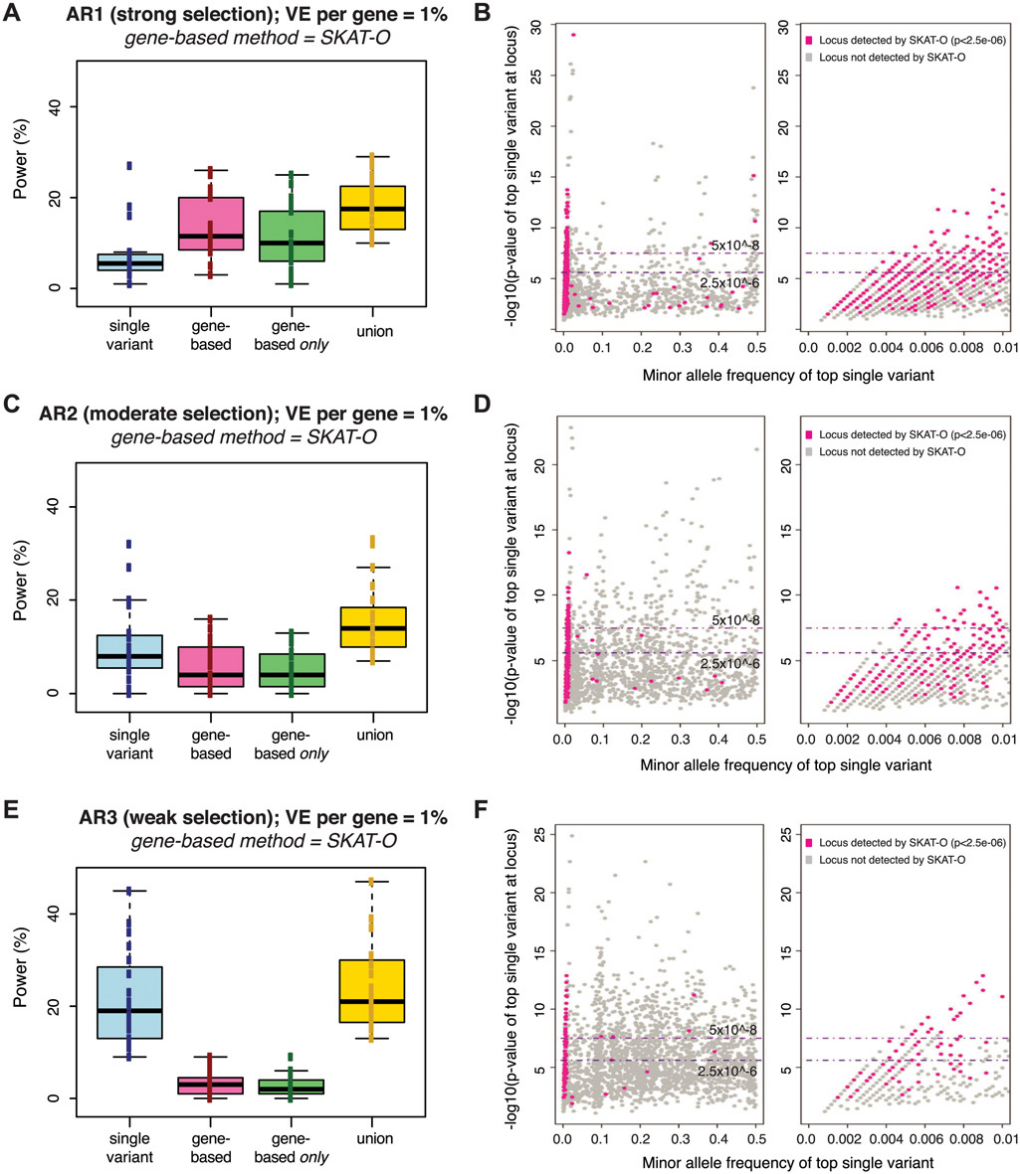
Power of best-performing gene-based rare variant method as compared to single variant association. Power is measured across one hundred simulations of phenotypic effects at each of 24 human gene loci in N = 3K samples (as in Fig 2). Under each architecture (AR1, AR2, AR3), the power of the best-performing gene-based test at alpha = 2.5e-06 (SKAT-O) is compared to single variant association (Fisher’s exact) at alpha = 5e-08 (panels A, C, E). No MAF threshold was applied to the single variant association tests; gene-based tests included only variants with MAF<1%. Blue boxplot shows range of power for single variant association across genes simulated; pink shows power of the gene-based test alone; green shows the fraction of loci detected only by gene-based test (and not single variant association); yellow shows the combined power of both gene-based and single variant association. Next to each boxplot (panels B, D, F) are scatterplots on which each simulated locus (under AR1, AR2, and AR3, respectively) is represented as a point based on the minor allele frequency (x-axis) and association p-value (y-axis) of the single most-associated variant (the top individual signal) across the locus. Single variant association detects loci plotted above the upper dotted line (at 5e-08), while gene-based association identifies a distinct subset of loci (those highlighted in pink, where the SKAT-O p-value is <2.5e-06). This latter group of loci are those where the top single variant is preferentially rare (and no common variant association signal exists); right-most scatterplots zoom into this portion of the x-axis (MAF<1%). Similar plots for AR4, AR5, and AR6 are shown in S10 Fig.

We next queried the overlap between signals detected by gene-based methods versus those detected by single variant association. In direct contrast to gene-based methods, the power of single variant association *decreased* as the contribution of rare variants *increased*: power at a genome-wide threshold of α = 5×10^-8^ for single variants was ~20%, ~10%, and ~7% under AR3, AR2, and AR1, respectively (blue bars in Fig 3A, 3C, and 3E). However, in all cases, the combined application of gene-based and single variant methods yielded greater sensitivity than single variant association alone (yellow bars in Figs 3A, 3C, 3E, and S10). This occurred because the association tests detect distinct subsets of loci: gene-based methods uniquely identified loci where the signal was driven by groups of rare variants for which single variant association test statistics were not individually significant (pink loci in Fig 3B, 3D, and 3F).

As expected, the comparative advantage of gene-based tests was most evident under architectures where there is strong purifying selection against causal alleles (under AR4, for example, the power of single-variant tests at α = 5×10^-8^ was <5%, while gene-based tests achieved ~50% power at α = 10^-4^, and ~20% power even at α = 2.5×10^-6^; S10A and S10B Fig). Under both AR2 and AR3 (where limited purifying selection made causal alleles more common), the power of single variant association (~20% at α = 5×10^-8^ under AR3) exceeded that of the best gene-based test (<5% at α = 2.5×10^-6^ under AR3), though each method detected unique loci. These results confirm that single variant and gene-based association methods should be jointly employed for maximal power across divergent locus architectures.

**Fig 4:**
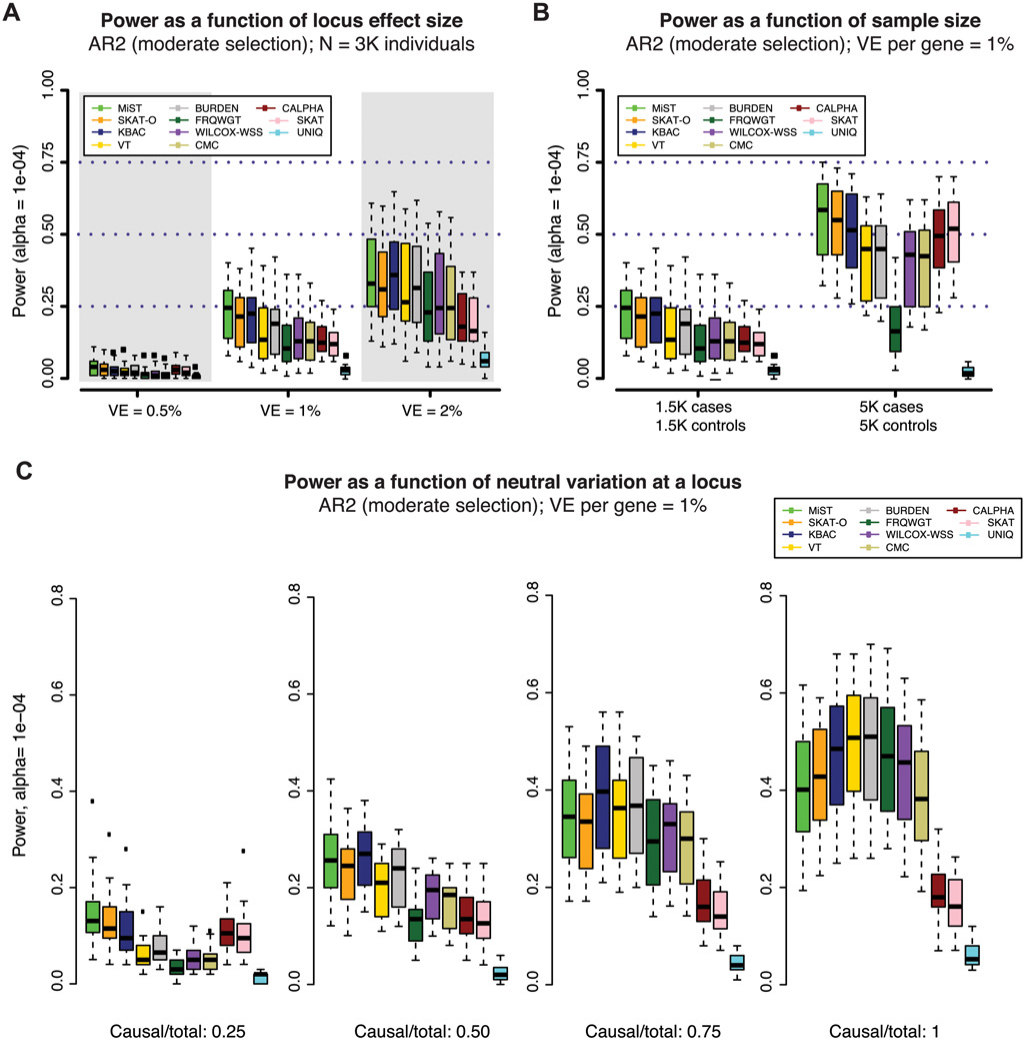
Power of gene-based methods as a function of sample size, locus effect size, and neutral variation. Power was measured across one hundred simulations at each of 24 gene loci (as in Figs 2 and 3). Across all panels above, variant effects were drawn from the architecture model AR2 (assuming moderate selection against causal variants, and thus modest inverse correlation between variant frequency and effect size). In **(A)**, variant effects were sampled at each locus such that the total fraction of phenotypic variance explained by the locus was ~0.5%, 1% (as in Figs 2 and 3) or 2%. In **(B)**, loci were simulated to explain 1% of phenotypic variance in sample sizes of 1.5K cases/1.5K controls (as in Figs 2 and 3) and 5K cases/5K controls. In both **(A)** and **(B)**, all exonic variants with MAF < 1% were included in the burden test (both causal and non-causal variants, resulting in a fewer than 50% of all tested variants being causal). In **(C)**, non-causal (neutral) variants were selectively removed such that the ratio of causal variants to total variants tested ranged from 0.25 to 1 (only causal variants tested). The gene-based methods each have varied performance under different locus effect sizes, sample sizes, and causal variant filtering scenarios.

To characterize the impact of locus effect size on the power of gene-based tests, we simulated loci where the phenotypic variance explained (VE) by genetic variants is 0.5%, 1% (as in Figs 2 and 3), and 2% (all under AR2). At loci where VE = 2%, power increased to nearly 40% (at α = 10^-4^), as compared to ~23% when VE = 1% (Figs 4A, S11A, and S11B). When VE = 0.5%, power was extremely low (<8% at α = 10^-4^ in 3K individuals), indicating that exome-wide sequencing studies of this size are substantially under-powered to interrogate genes for weaker effects (S11A Fig).

### Impact of sample size and neutral variation on power of gene-based association

The relatively modest power of gene-based tests at stringent levels of significance across the architectures considered here presents challenges to investigators seeking to discover novel disease-associated loci in studies of this size. Thus, we next investigated the extent to which power could be improved by a) increasing sample size, or b) excluding neutral variation at a locus.

We found that gene-based methods exhibit differential gains in power as sample size increases from 3K to 10K individuals (Fig 4B). The median power of MiST, for example, increased from ~23% to ~60% (at α = 10^-4^, under AR2) in 10K samples and was largely retained (~50%) even at α = 2.5×10^-6^ (S11C Fig). However, the increase in power was not uniform across methods. This occurred, in part, because (unlike for single variant tests) the relationship between sample size and power is not straightforward for gene-based tests: as sample size increases, causal alleles are observed more times, but the number of (rare) *non-causal* alleles also grows sharply. Thus, methods that up-weight all rare alleles regardless of their observed effect (e.g., FRQWGT) may benefit least from increases in sample size (S11–S13 Fig).

As the number of observations of rare alleles increases with sample size, the performance of single variant association tests will certainly improve, but our analysis suggests that gene-based tests will still uniquely identify loci at which the aggregate signal is driven by variants too rare to be individually detected. When the top single variant in our simulated datasets had MAF < = 0.4%, the locus was rarely detected by single variant association in a sample of 3K individuals (Fig 3B, 3D, and 3F). Single variant tests would have <80% power to detect an effect at a variant of that frequency (at α = 5×10^-8^) even in 10K samples, unless the RR of that variant was over 3. Moreover, as sample sizes increase, the threshold required to assess significance for gene-based methods will remain the same (as the number of independent tests performed will not change), while that for single variant association tests will need to become more stringent as more novel variants are discovered. Hence, we expect the joint application of single variant and gene-based methods to remain beneficial even as sample sizes increase.

Our study also confirmed that gene-based tests are highly sensitive to the fraction of neutral variation at a locus (Figs 4C and S13), as has been previously described [10, 11, 23]. We additionally found that unidirectional burden tests exhibit the sharpest increases in power as the fraction of neutral variation decreases. Under AR2 in 3K individuals, KBAC power at α = 10^-4^ exceeded 50% when only disease-causing variants were included (increasing from ~22% prior to variant filtering). These tests may therefore be most powerful for testing targeted hypotheses at loci where rich functional annotation enables exclusion of a subset of neutral variants. Conversely, variance-component tests (C-ALPHA, SKAT) as well as combined methods (MiST, SKAT-O) are characterized by a relative immunity to neutral variation. This latter group of methods, then, are attractive options for jointly testing large numbers of less strictly filtered variants (e.g. in a pathway-based analysis).

### Concordance between the results of different gene-based association methods

**Fig 5:**
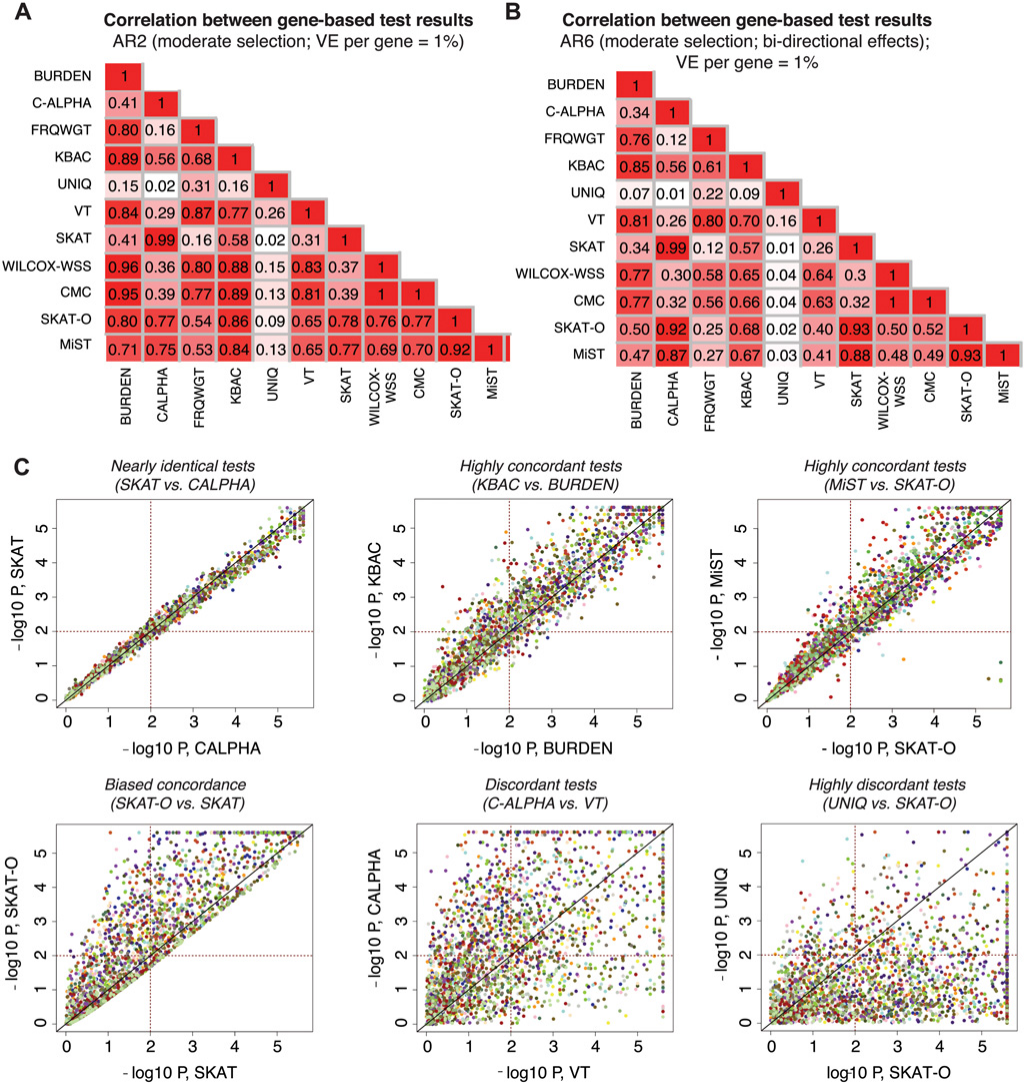
Concordance between results of different gene-based methods. Pairwise correlation coefficients (R2) between the p-values reported by different gene-based association methods under AR2 (moderate selection; shown in **(A**) and under AR6 (moderate selection and bi-directional phenotypic effects, shown in (**B)**). P-values above 0.1 are excluded in computation of the correlation. In (**C**), scatter plots show the results (-log10 of the p-values) reported by a pair of gene-based tests under AR2; p-values below 5e-06 are plotted at 5e-06. Each point represents an individual locus at which both gene-based methods were applied (2400 total points); points of the same color represent different simulations at the same gene loci (e.g. same gene and haplotype structure, but different variant phenotypic effects). Dotted lines mark p = 0.01, such that points above the horizontal line or to the right of the vertical line represent loci at which nominally significant results are reported by the gene-based method. All data above generated in 3K samples (1.5K cases, 1.5K controls).

We next investigated the degree of overlap between signals detected by each gene-based method. For each pair of association methods, we computed Pearson’s correlation coefficients between their reported p-values on a logarithmic scale (Figs 5A, 5B, and S14). We found that tests with similar design characteristics (e.g., SKAT and C-ALPHA, R^2^ = 0.99) exhibit very high correlation, as expected (Fig 5C). Some methods were highly correlated, but there was variability in the p-values reported (e.g., MiST and SKAT-O, R^2^ = 0.92), while others were much less related or even uncorrelated (e.g., SKAT-O and UNIQ, R^2^ = 0.02). While in this latter case low correlation was driven by the lower mean power of UNIQ relative to SKAT-O, it is worth noting that there did exist a set of true causal loci (where many case-private singletons segregate) at which UNIQ reported p<10^-4^, but SKAT-O reported p>0.01 (Fig 5C).

Other methods, such as SKAT and SKAT-O, showed asymmetric concordance (R^2^ = 0.78): SKAT-O detected a set of causal loci entirely undetected by SKAT, but was more conservative on the whole, reporting p-values up to an order of magnitude higher than those reported by SKAT at the majority of loci tested. These correlations were also architecture-dependent: under AR2 (where there are only deleterious effects), for example, SKAT-O exhibited high concordance with KBAC (R^2^ = 0.86), while under AR6 (where bidirectional effects are present), SKAT-O was most concordant with C-ALPHA and SKAT (R^2^ = 0.93). MiST shared this behavior, reflecting the ‘unified’ design of these tests as combinations of a unidirectional burden test and a bidirectional variance-based method [23, 24].

**Fig 6:**
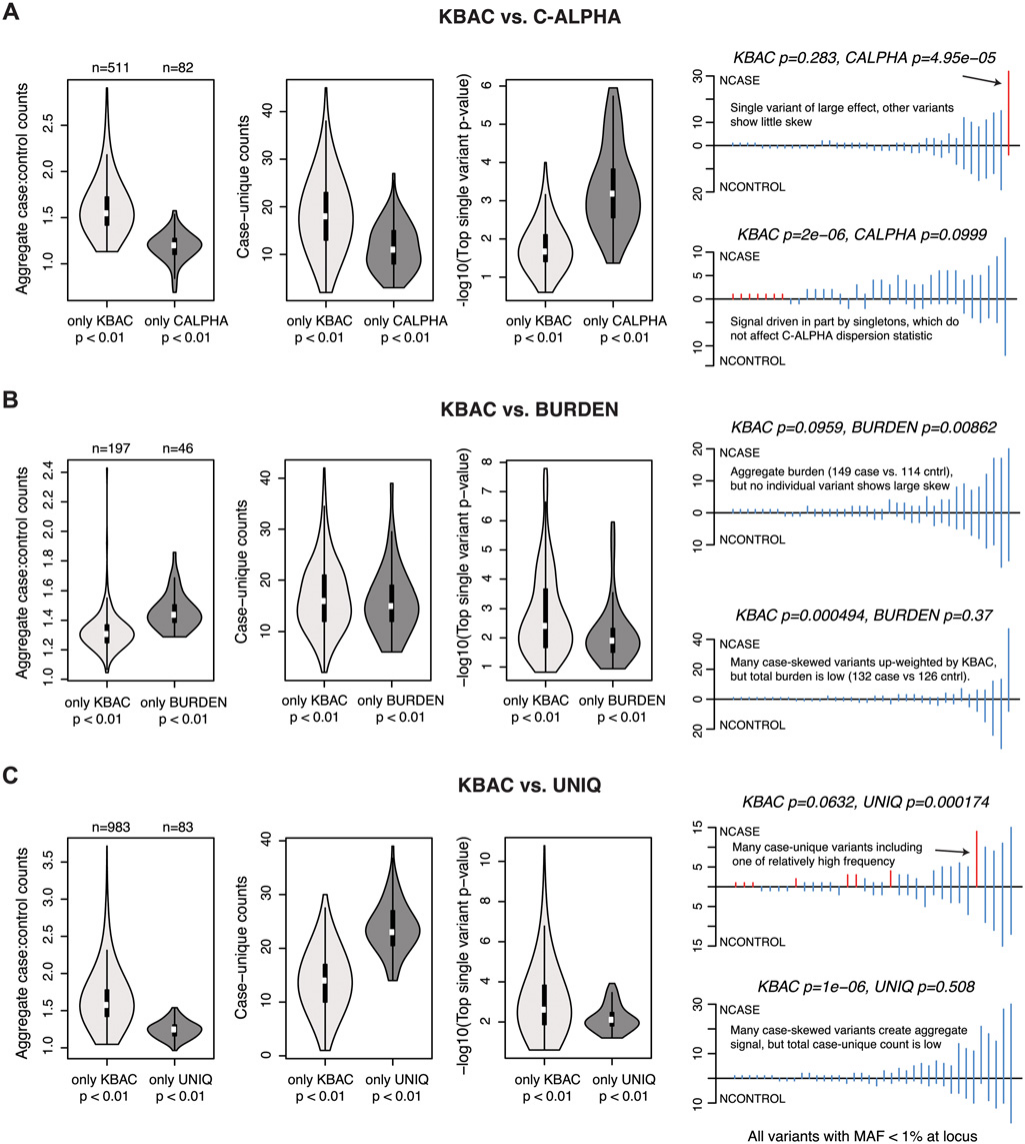
Properties of loci at which gene-based methods report discordant results. Characteristics of causal loci at which KBAC (the method with highest mean power at nominal levels of significance) produces discordant results as compared to another gene-based method. Results are shown above for the simulated architecture AR2 in 3K samples. KBAC is compared to the **(A)** C-ALPHA, **(B)** BURDEN, and **(C)** UNIQ gene-based methods. In each comparison, loci are identified at which KBAC (but not the other method) reports a p-value < 0.01, or at which the other method (but not KBAC) reports a p-value < 0.01. For each group of loci, leftmost vioplot shows the distribution of aggregate case:control counts (number of minor alleles observed in cases divided by number of minor alleles observed in controls, for variants with MAF<1%). Middle vioplot shows distribution of case-unique counts (number of observations of alleles that are only present in cases and absent from controls). Rightmost vioplot shows distribution of the top single variant p-value observed for an exonic variant at the locus (log10 scale). Line plots at right show the distribution of variants (MAF < 1%) at representative simulated loci where the methods are discordant. Each line represents a variant; height above line measures the variant’s case counts, while height below measures control counts. Red lines highlight variants which drive the difference in test performance.

To understand the drivers of such differences and identify scenarios where certain tests may be more powerful than others, we conducted pairwise comparisons between KBAC (one of the highest performing methods at α = 10^-4^ across AR1-AR5) and the other gene-based methods. We focused here on loci where VE = 1%, simulated under AR2. For each comparison, we characterized the properties of loci at which KBAC (but not the other method) reports *p*<0.01, and vice-versa. In the comparison between KBAC and C-ALPHA (Fig 6A), we found that loci at which only KBAC detected signal were characterized by a higher aggregate skew in case to control counts (often driven by singletons, which do not contribute to the variance component tests’ dispersion statistic). Loci at which only C-ALPHA detected signal, on the other hand, were characterized by a relatively common single variant of large effect (in the background of many variants with balanced case to control counts).

For loci where the ratio of aggregate case to control counts is high, but no individual variants/genotypes show any substantial skew, the BURDEN test may be more powerful than KBAC (Fig 6B). This makes sense: KBAC adaptively weights multi-site genotype counts by their observed case-bias, and if all variants have low weights, the maximum achievable KBAC statistic is low, whereas BURDEN quantifies the significance of the observed signal in *aggregate*. Finally, UNIQ (unsurprisingly) more readily detected loci at which signal is driven by either many rare variants private to cases, or by a single relatively frequent case-unique (or control-unique) variant (Fig 6C). Taken together, these data indicate that although a given method may exhibit high *mean* power across divergent architectures, it may not be optimal for testing specific genetic hypotheses.

Given the observation that different methods capture different signals, we wondered whether a strategy in which subsets of methods are collectively applied to a locus might be informative in an exome-wide setting (e.g., to test multiple hypotheses about locus architecture at once). To test this, we employed a stepwise forward selection approach, starting with each of the three best-performing gene-based methods across architectures (MiST, SKAT-O and KBAC) and using the degree of difference (in orders of magnitude) between additional methods’ reported p-values as the inclusion criterion (see Methods, S1 Text).

In 3K individuals, under AR2 (where MiST power is ~23% at α = 10^-4^), we found that particular combinations of tests (e.g., KBAC+MiST+VT+UNIQ+FRQWGT) could jointly achieve ~31% sensitivity at α = 10^-4^ (using the single minimum p-value reported across all three tests). However, this gain came at the cost of a higher false positive rate (FPR): after adjusting the p-value significance threshold to correct for the increase in FPR, we found negligible gains in power compared to the application of a single test (S3 Table). Joint application of gene-based tests may still be useful, however, in settings where a higher FPR is tolerable, e.g., to increase sensitivity in a ‘discovery’ exome-wide sequencing scan which precedes large-scale targeted follow-up.

## Discussion

Given the wide array of aggregate rare variant association methods now available for application in re-sequencing or genotyping studies of complex traits [30], it is critical to characterize and quantify the statistical power of each method to test heterogeneous genetic hypotheses. In this study, we conducted a comparative analysis of a panel of commonly used gene-based rare variant association tests under a broad range of realistic allelic architectures, significance thresholds, locus effect sizes, sample sizes, and filters for neutral variation.

In sample sizes comparable to those of many contemporary sequencing studies (3K case-control individuals), we find that while gene-based association methods augment the power of single variant tests by preferentially detecting loci at which rare variants drive the causal architecture, their absolute power is low. All gene-based methods evaluated in this study have limited power, even to detect loci explaining as much as 1% of the variance in phenotypic liability underlying a common trait such as type 2 diabetes (mean power across architectures is ~5–20% at α = 2.5×10^-6^). Even in 10K case-control samples, power remains modest (~60% at α = 2.5×10^-6^). Based on estimates of variance explained by known rare and common variant signals (the strongest single common variant association for T2D, mapping near *TCF7L2*, explains ~1% of phenotypic variance), it seems probable that for any given complex trait, at best a handful of loci will have effects on this scale. The full potential of exome sequencing to provide biological insights into disease, then, will depend largely on the detection of loci of smaller aggregate effects, and will require far larger sample sizes than these.

The low mean power to detect disease-associated loci prompted the question of whether some methods are better powered than others to discover novel signals under specific hypothesized locus architectures. We find that at more stringent significance thresholds (α<10^-4^), MiST and SKAT-O have the highest power across architectures simulated here, especially when rare variants have bidirectional effects on disease. Thus, for investigators looking to discover signals across thousands of loci (e.g., in exome-wide scans), these tests are likely to maximize sensitivity.

Weighted sum methods (and KBAC in particular), on the other hand, are consistently best-powered to detect rare variants of deleterious effect at less stringent levels of significance, and also show the greatest gains in power when neutral variation can be filtered out. These attributes may be useful in various scenarios: to test a small number of biological hypotheses (e.g. at only a few loci, especially if functional annotations are available), to prioritize signals for further follow-up from a discovery scan, or to place bounds (e.g., after an exome-wide sequencing study) on the total number of genes harboring rare variants of a given effect size that are likely to exist.

In addition to MiST, SKAT-O and KBAC, we find that other methods may have individual strengths under particular scenarios (e.g., UNIQ to test whether a gene harbors an excess of highly penetrant rare variants, or BURDEN to detect a collection of variants each of very weak effect); these methods may be optimal for testing such specific genetic hypotheses. Finally, in larger sample sizes (n = 10K case-control individuals), our simulations demonstrate that the increasing number of neutral (non-causal) rare variants may limit gains in the power of some methods (e.g. FRQWGT). Here, MiST is best-powered at stringent significance thresholds. Taken together, these results suggest that the interpretation of novel signal discovery (or the lack thereof) in sequencing studies may vary based on the specific gene-based methods that are used.

This study has a number of limitations. It is based on simulated data (albeit data consistent with available empirical information on genetic variation and disease epidemiology [15]). It does not explore the effects of properties such as demographic history, gene size, mutation rate, haplotype length, or degree of linkage disequilibrium between causal variants on the power of gene-based association methods. Moreover, it does not characterize the performance of these methods at non-coding regions, where causal variant frequencies and effect sizes may be different, and where there is likely a higher proportion of neutral variation. This simulation approach, however, enabled us to undertake a controlled, quantitative characterization of the performance of gene-based association methods under a range of scenarios. Future work should characterize these methods in study populations of different ethnicities, where different site frequency spectra and linkage disequilibrium patterns between causal variants may alter power (S9 Fig). Architectures we simulated assumed a common binary trait; power to detect loci explaining phenotypic variance for less prevalent traits is likely higher, but we did not study this relationship. The tools available on our website (http://mccarthy.well.ox.ac.uk/publications/2014/moutsianas_simulations/) allow the investigation of this question for any complex trait by generating simulated data using a custom, user-specified RR-by-allele frequency heat-map and disease prevalence.

In summary, we find that specific gene-based association methods are best deployed in the setting of particular experimental study designs, and when testing for particular genetic models of disease. Such an approach will likely enable meaningful interpretation of both positive and negative findings in ongoing sequencing studies, and is bound to remain important even as sample sizes increase and new statistical methods for aggregate testing of rare variants are developed.

## Methods

### Generation of simulated reference panels

Simulated datasets were generated using HAPGEN2 [12]. HAPGEN2 generates case-control data using a haplotype reshuffling approach based on the Li & Stephens model [31]. Under this model, simulated (unobserved) haplotypes are assumed to be an imperfect mosaic of actual (observed) haplotypes and are simulated using a Hidden Markov Model with recombination and mutation rates as parameters. Case and control samples are generated by over-sampling haplotype segments which contain alleles at which phenotypic effects are introduced (based on the relative risks assigned to them). A phased reference panel of haplotypes from 379 European (98 TSI, 89 GBR, 85 CEU, 14 IBS, and 93 FIN) individuals from the 1000 Genomes Project (1000G Project Phase 1, release 3) [13] was augmented to 12,514 individuals by iteratively simulating haplotypes (with no phenotypic effects) and adding them to the original reference panel, in increments of 300 individuals per iteration.

An excess of rare variation was introduced to the data using an empirically selected value of θ = 0.08 for the mutation parameter in HAPGEN2, so as to match the singleton count observed in empirical re-sequencing data in a sample of this size. We used the SFS reported by Nelson *et al* [14], which was based on sequencing 351kb of coding sequence in 12,514 samples of European descent. The resulting dataset was subsequently thinned using a rejection sampling approach, to match the full site frequency spectrum observed in real data. This two-step approach (matching for singletons, and then thinning the dataset) was necessary to model the excess in rarer variation observed in whole exome sequencing datasets while preserving the LD structure of the reference panel. In order to validate that this approach led to a realistic SFS when sub-sampled to smaller sizes, we compared the SFS observed in the simulated, thinned panel, in subsets of 2,738 individuals, to that of empirical exome-wide sequencing data on the same number of individuals, from the GoT2D project (dark and light blue lines, Fig 1).

### Forward simulation of population-scale data to model genetic architecture

Forward population genetic simulations of global complex disease architecture (specifically, for type 2 diabetes, a disease of prevalence 8% and heritability ~45%) were conducted across a range of disease models varying in mutational target size and coupling to purifying selection [15]. By varying only these two parameters, a wide range of continuous joint frequency and effect size distributions were generated; under models with strong coupling to selection, rare variants explain the bulk of heritability and have large effects, while under models with weak coupling to selection, common variants explain the bulk of heritability and rare variants have weaker effects. For the HAPGEN2 simulations conducted here, we sampled variant effect sizes from the distributions observed in the forward simulated datasets at loci explaining ~1% of phenotypic variance underlying T2D (S3 Fig).

### Assignment of variant phenotypic effects within HAPGEN2 simulations

Variant effects were selected from the frequency-effect size distributions described above. We simulated these effects at randomly selected exonic variants across each gene. We used variant frequencies measured in the augmented reference panel of 12,514 individuals. In unidirectional architectures, all rare variants were assumed to increase risk of disease (RR>1). In bidirectional architectures, protective effects were sampled in the same way, but the relative risks were inverted. Variant effects were sampled until the cumulative variance explained (VE) on the liability scale by each locus reached the desired threshold (e.g. VE = 0.5%, 1%, or 2%). The following procedure was followed for introducing variation at each locus:

- Pick an exonic variant at random
- Introduce an effect by sampling from the frequency-RR distribution of the respective architecture
- If the cumulative variance explained (on the liability scale, %VE) by variants at the locus is below of the specified threshold, go to step (i) and repeat
- If the variance is above the specified threshold, remove one of the introduced effects (at random) and go to step (i)
- If the cumulative variance explained is close enough to the specified threshold (0.95*VE,1.05*VE), then
  - If the number of introduced variants is over 35, quit and restart, else:
  - Accept the sampling and simulate data using the variants and effect sizes chosen, using HAPGEN2.

The upper bound of 35 on the total number of causal variants introduced per locus was imposed due to instability in HAPGEN2 behavior above this threshold; this limit was rarely reached in 3K samples, but it did restrict architectures simulated in 10K samples (S11C and S12 Figs).

The calculation of variance explained at each locus was conducted using the method described by So et al., which is available online as an R script [26]. This calculation requires three parameters as input (per variant): the prevalence of the trait (in this case assumed to be 8%, to model type 2 diabetes), the population frequency of the risk allele, and the genotype relative risk. We assumed independence between risk variants at a given locus, and thus estimated the total percentage of variance explained as the sum of the variance explained by each individual variant.

### Running gene-based tests of association on simulated data

The latest releases of the PLINK/SEQ (v0.09) [18] and EPACTS (v3.2.3) [25] software packages were used to run ten of the gene-based methods evaluated in this study. MiST was run using a publicly available R package (http://cran.r-project.org/web/packages/MiST/index.html) [24]. All exonic variants (causal and non-causal) below varying minor allele frequency thresholds (1% for all analyses discussed in the main text, unless otherwise stated) were included in the tests, except when the fraction of neutral variation was varied. In this case, the proportion of causal variants included in the test was fixed to 0.25, 0.50, 0.75, or 1 (Fig 4C).

### Selecting a subset of tests for joint application to the data

The subsets of tests chosen for inclusion into composite tests were selected using a stepwise forward selection approach. Starting with a single test (three runs per architecture, each starting with one of the top three performing tests across architectures, MiST, SKAT-O and KBAC), the next test to be included at each step was the one which reported the greatest number of novel signals, *i*.*e*. not previously detected by the tests already included. Novel signals were defined as loci for which the p-value reported by the candidate test for inclusion was lower by a specified multiplicative “margin” (factor) than the lowest p-value reported by tests already included in the composite test. Three margins were used (100, 10, and 1); a margin of 100, for example, implies that for signals to be considered novel, they p-value of the candidate test needs to be two orders of magnitude lower than the lowest of the ones already included in the composite test.

### Datasets and software

All datasets discussed in this study, together with the scripts used to generate them and results of both single variant association and gene-based methods across all architectures, are available on the website http://mccarthy.well.ox.ac.uk/publications/2014/moutsianas_simulations/. The website also contains the software used for the script generation (a wrapper for HAPGEN2 [12]), which can be used to generate analogous simulated data for the genes we included in the manuscript under alternative scenarios/architectures.

## Supporting Information

S1 Text List of all supporting tables and figures; supplemental methods (this file). (PDF)

S1 Fig Pairwise linkage disequilibrium between variants in simulated vs. empirical data, for different minor allele frequency categories. Mean pairwise LD between variants (measured by r^2^) as a function of the distance between each pair of variants. The pairwise r is shown for the empirical 1000 Genomes reference panel used in this study (red lines) and variants in the simulated panel which was expanded using HAPGEN2 (blue lines; see S1 Text for details about expansion). Each plot shows variants split by minor allele frequency category; in all cases, linkage in the simulated data mimic what is seen in empirical data. Data is shown at a single representative gene locus (GATA3). The mean pairwise LD was calculated and averaged across 10 different subsets (each containing 379 samples, to match the size of the 1000 Genomes European reference panel) of data simulated using HAPGEN2 at the GATA3 locus.
(PDF)

S2 Fig Comparison of site frequency spectrum at 202 genes in Nelson et al vs. all other REFSEQ genes. All simulations discussed in this manuscript were conducted (in HAPGEN2) to match the empirical site frequency spectrum reported across 202 genes in Nelson et al (Science, 2012; main text reference 14). This comparator dataset was chosen because of its large sample size (12K individuals). To confirm that the site frequency spectrum across these 202 genes (‘Nelson et al gene set’) is representative of the distribution across all genes, we compared the SFS at these genes to that observed for all other genes in REFSEQ across the genome (‘All other genes’) in a dataset of 2,657 European individuals who were whole-exome sequenced by the Go-T2D Consortium. These data confirm that the Nelson et al genes are not outliers on the basis of observed minor allele counts.
(PDF)

S3 Fig Variant frequency-effect size distributions under each simulated architecture. The below frequency-RR distributions were learned from population genetic forward simulations of global genetic architecture (Agarwala et al, Nature Genetics 2013). (**A**) AR1 assumes strong coupling to purifying selection; that is, variants under selection (more likely rare) have larger effects on disease. (**B**) AR2 assumes moderate coupling to selection, and (**C**) AR3 assumes no coupling to selection (thus effect sizes are more uniform across the frequency spectrum). Figures on the right are zoomed-in versions of those on the left (only showing variants with MAF up to 1%).
(PDF)

S4 Fig Distribution of number of causal variants and total number of simulated variants tested per locus under different architectures. All results shown below are for loci which explain 1% of phenotypic variance, simulated in 3K samples (1.5 cases / 1.5K controls). In (**A**) and (**B**) variant counts are shown for the simulated architecture AR2 (moderate selection). In (**A**) is shown a histogram of simulated loci, binned by the number of causal variants sampled per locus in order for the locus to explain 1% of phenotypic variance. In (**B**) is shown a histogram of simulated loci, binned now by the total number of exonic variants with MAF<1%, e.g. the total number of variants included in gene-based association testing. In (**C**), distributions of variant counts (for both causal variants and the total number of variants tested per locus) are shown as box plots, under all six simulated architectures (S2 Table).
(PDF)

S5 Fig Power of gene-based tests under null locus architectures to assess type I error. All gene-based tests were relatively well-calibrated and had expected type I error rates at both **(A**) alpha = 0.05 and (**B**) alpha = 1e-04. Some tests, such as SKAT, appear to be relatively conservative (as has been previously described).
(PDF)

S6 Fig Relative power of gene-based tests using an absolute significance threshold vs. an empirical threshold corrected for the false positive rate of each test. All results shown below are for loci which explain 1% of phenotypic variance, simulated in 3K samples (1.5 cases / 1.5K controls). Results in (**A**) were simulated under architecture AR2 (moderate selection, unidirectional effects); results in (**B**) were simulated under AR6 (moderate selection, bidirectional effects). FPR-corrected power is calculated using an empirically-derived threshold at which the observed false positive rate is 1e-04; this threshold varies for each gene-based method based on how conservative each method is (this variability is shown in S8 Fig). The relative power of the highest-ranked methods is unchanged with corrected vs. uncorrected significance thresholds.
(PDF)

S7 Fig Power of gene-based tests in 3K samples, as a function of significance threshold, under each simulated architecture. Causal variants at each simulated gene explain 1% of phenotypic variance. In (**A**), architectures were simulated with causal variants spanning the full frequency spectrum (including common alleles), and all causal alleles increase risk of disease. Gene-based association testing was performed only on variants with MAF < 1%. In (**B**), only variants with MAF<1% are causal (thus rare alleles are responsible for the entire locus contribution to heritability). In (**C**), variants across the full site frequency spectrum are causal, but causal alleles are mixed in direction of effect; some increase risk of disease, while others reduce risk of disease.
(PDF)

S8 Fig Power of gene-based methods in 3K samples using different minor allele frequency thresholds for burden testing. Causal variants at each simulated gene explain 1% of phenotypic variance (contributed by variants across the full frequency spectrum). In Figs 2 and 3 of the main manuscript, a MAF threshold of 1% is used for inclusion of variants into the gene-based association test. Shown here are power results using a MAF threshold of 0.5% and 1%. All simulations below were conducted under AR2 (moderate selection against causal alleles), for loci explaining 1% of phenotypic variance, and in 3K samples.
(PDF)

S9 Fig Effect of linkage disequilibrium between causal variants on power of gene-based tests. Each boxplot below shows the range of p-values reported by each gene-based method for loci binned by the average pairwise linkage disequilibrium (LD; as measured by r^2^). Across all the gene-based tests evaluated here, the mean p-values are lower (i.e., more significant) when the extent of LD between causal variants at the locus is low.
(PDF)

S10 Fig Power of gene-based method (SKAT-O) as compared to single variant association testing under AR4, AR5, and AR6. Power is measured across one hundred simulations of phenotypic effects at each of 24 human gene loci in N = 3K samples. Under each architecture (AR4, AR5, AR6), the power of one of the best-performing gene-based tests (SKAT-O) at alpha = 2.5e-06 is compared to single variant association (**A**,**C**,**E**). The significance threshold used for the gene-based test is 2.5e-06; the threshold for single variant association (Fisher’s exact) is 5e-08. Blue boxplot shows range of power for single variant association across genes simulated; pink shows power of the gene-based test; green shows the fraction of loci detected only by the gene-based test (and not single variant association); yellow shows the combined sensitivity of both gene-based and single variant association. Next to each boxplot (panels **B**,**D**,**F**) are scatterplots showing the distinct sets of loci detected by single variant association (loci above the upper dotted red line at 5e-08) and by gene-based association (highlighted in orange). Loci are plotted based on the minor allele frequency (x-axis) and association p-value (y-axis) of the most associated single variant across the locus. Similar plots for AR1, AR2, and AR3 are shown in Fig 3 of the main manuscript.
(PDF)

S11 Fig Power of gene-based tests as a function of locus effect size and sample size. Power is shown here under AR2 (moderate coupling to selection) for varying locus effect sizes and sample sizes. (**A**) VE = 0.5%, per locus, N = 3K samples, (**B**) VE = 2%, N = 3K samples, (**C**) VE = 1%, 10K samples. In Fig 2 of the main manuscript, data was shown for VE = 1% and N = 3K individuals, across a range of architectures.
(PDF)

S12 Fig Effect of increasing sample size on the simulated number of causal and total segregating variants, and effect of cap on number of causal variants in HAPGEN2. We simulated loci at which the site frequency spectrum (and thus the total number of segregating variants) matched empirical datasets (see main manuscript Fig 1). At these loci, causal variants were sampled from different frequency-effect size distributions; due to technical limitations of HAPGEN2, a maximum of 35 causal variants were selected to collectively explain 1% of phenotypic variance. As this figure demonstrates, this cap did not materially impact simulations in 3K samples, but in 10K samples, this variant cap does restrict the diversity of locus architectures that we were able to simulate. In 3K samples, the median total number of segregating variants with MAF<1% per locus is ~38; ~18 of these variants (well below 35) have causal effects on disease. In 10K samples, the ratio of causal to total variants at simulated loci is substantially reduced due to the cap on the number of causal variants. (**A**) The number of total segregating variants (red) and the number of variants simulated to have causal effects (blue) per locus. (**B**) The distribution of number of causal variants per locus in 10K samples with and without the variant cap. We only simulated loci with fewer than 35 causal variants (pink distribution).
(PDF)

S13 Fig Power of gene-based methods in 3K vs. 10K samples, as a function of ratio of causal to total number of variants per locus. All results shown in this figure are for loci that explain 1% of phenotypic variance; gene-based tests run in 3K samples (1.5 cases / 1.5K controls) or in 10K samples (5K cases / 5K controls); loci simulated under AR2 (moderate selection).
(**A**–**B**) Each point represents a simulated locus (across 24 human genes, 100 replicates per gene, simulations in both 3K and 10K samples; 4800 points are plotted in each scatterplot). The x-axis shows the ratio of number of causal variants (variants to which phenotypic effects are assigned) relative to total number of variants tested across the locus (all variants with MAF<1%). The y-axis shows p-values reported by the gene-based association method SKAT-O. All loci simulated in both 3K and 10K samples are shown as grey points in both panels (**A**) and (**B**). Red points are loci simulated in 3K samples; blue points are loci simulated in 10K samples. Blue points (loci simulated in 10K samples) are left shifted relative to red points (loci simulated in 3K samples) because the fraction of neutral variation increases with sample size in these simulations. Blue points are also shifted up, as p-values in 10K samples are more significant than those in 3K samples. In both sample sizes, SKAT-O p-values are more significant as the ratio of causal to total number of variants increases.
(**C**–**D**) Mean p-values reported by two gene-based association methods (SKAT-O and FRQWGT) as a function of the proportion of causal variation at a locus, in 3K (**C**) and 10K (**D**) samples. In 3K samples, both methods show similar performance, but in 10K samples SKAT-O significantly outperforms FRQWGT method, regardless of the proportion of causal variation. While the lower mean power of FRQWGT in 10K samples may be partially attributable to the larger number of neutral variants in this sample size (S12 Fig), panel (**D**) suggests that FRQWGT may also be less-powered for other reasons (and is not entirely a result of bias introduced by the cap on the number of causal variants in HAPGEN2). This underscores the need to select well-powered gene-based methods for association testing in large sample sizes.
(**E**) Power of the gene-based method MiST at alpha = 1e-04 in 3K and 10K samples, as a function of the ratio of causal to total number of variants per locus. Regardless of this ratio, power of MiST is substantially higher in 10K samples (blue) as compared to 3K samples. Power in both sample sizes increases as the proportion of causal variation increases, but power in 3K samples is more sensitive to this property than is power in 10K samples. In 10K samples, power remains ~60–70%, regardless of the proportion of causal variation. This further suggests that the causal variant cap in HAPGEN2, while restrictive, does not dramatically affect characterization of gene-based test power in 10K samples.
(PDF)

S14 Fig Concordance between p-values reported by different gene-based association methods under each simulated architecture. All results shown here are for loci which explain 1% of phenotypic variance; gene-based tests run in 3K samples (1.5 cases / 1.5K controls). Values represent R^2^ correlation coefficients between p-values reported by each gene-based association method. Correlation coefficients are shown under all simulated architectures: **(A**) AR1, (**B**) AR2, (**C**) AR3, (**D**) AR4, (**E**) AR5, and (**F**) AR6.
(PDF)

S1 Table List of human gene loci at which HAPGEN2 simulations were performed. 24 genes on chr10 were selected from the center of the distribution of human gene coding length (Fig 1). Below are the genomic locations of these regions; HAPGEN2 simulations were performed across the full length of each transcript. Causal variants were selected from exonic regions only, and burden testing was also run on variants (causal and non-causal) within the exonic regions only.
(XLSX)

S2 Table Locus architectures modeled at simulated loci. The below range of locus architectures were modeled at simulated loci; variant effect sizes were sampled from joint frequency-effect size distributions learned from forward population genetic simulations (Agarwala et al, Nature Genetics 2013). The architectures were chosen to reflect a range of different rare variant contributions and effect sizes. Briefly, the ‘selection parameter’ controls the degree to which the effect of a variant on evolutionary fitness is coupled to its effect on disease. When tau = 1, variants that are deleterious for fitness (and thus rarer in the population) have the largest effects on disease. When tau = 0, all causal variants have comparable additive effects on disease regardless of their effects on fitness, and thus common causal variants contribute a greater proportion of total heritability (S1 Text). At each locus, the total number of causal variants depended on the effect sizes sampled, as loci were modeled to explain a fixed proportion of liability-scale phenotypic variance underlying a complex trait with 8% prevalence (such as type 2 diabetes).
(PDF)

S3 Table Power of ‘composite’ groups of gene-based association methods. Test combinations were picked using step-wise forward selection starting from each of the three best-performing gene-based association methods across architectures (KBAC, SKAT-O, MiST). First column lists the architecture (see S2 Table and main text for more information). VE refers to total phenotypic variance explained by the locus; D/M describes whether causal effects are deleterious only (D) or a mix of deleterious and protective (M). The second column contains the ‘starting’ test, and the third column indicates the ‘margin’ of difference in p-values used in the forward selection algorithm (where 100 is 2 orders of magnitude, 10 is 1 order of magnitude and 1 is minimum margin; see S1 Text). The fourth column contains the list of tests picked by the selection algorithm until no other addition offered higher power under that margin, in the order of selection. Column 5 shows the total sensitivity of the composite test (when the minimum p-value across all tests in the group is used), using an (un-adjusted) p-value threshold of 1e-04. Column 6 shows power under the adjusted p-value threshold (to maintain a false positive rate < = 1e-04). Column 7 shows power of the single best gene-based test for comparison (at alpha = 1e-04). For each architecture, the best-performing ‘composite’ test is shown in bold.
(PDF)
