# Supplementary material for: The Power of Gene-Based Rare Variant Methods to Detect Disease-Associated Variation and Test Hypotheses About Complex Disease
Source: PLoS Genet. 2015 Apr 23;11(4):e1005165. doi: 10.1371/journal.pgen.1005165 (PMC4407972; doi:10.1371/journal.pgen.1005165)
Supplement: S1 Text — (PDF) [file pgen.1005165.s001.pdf]

S1 Text

The Power of Gene-based Rare Variant Methods to Detect Disease-associated Variation and Test Hypotheses about Complex Disease

Loukas Moutsianas[1,*] , Vineeta Agarwala[2-3,*], Christian Fuchsberger[4], Jason Flannick[3,5], Manuel A. Rivas[1], Patrick K. Albers[1], Kyle J. Gaulton[1], the GoT2D Consortium[6], Gil McVean[1], Michael Boehnke[4], David Altshuler[3,5,7,8], Mark I. McCarthy[1,9]

1. Wellcome Trust Centre for Human Genetics, University of Oxford, Oxford OX3 7BN, UK
2. Program in Biophysics, Harvard University, Cambridge, MA 02138, USA
3. Program in Medical and Population Genetics, Broad Institute of Harvard and MIT, Cambridge, MA 02142, USA
4. Department of Biostatistics and Center for Statistical Genetics, University of Michigan, Ann Arbor, MI 48109, USA
5. Center for Human Genetic Research, Massachusetts General Hospital, Boston, MA 02114, USA
6. A full list of GoT2D members and affiliations appears below.
7. Department of Genetics, Harvard Medical School, Boston, MA 02115, USA
8. Department of Biology, Massachusetts Institute of Technology, Cambridge, MA 02139, USA
9. Oxford Centre for Diabetes, Endocrinology and Metabolism, University of Oxford, Oxford OX3 7LJ, UK

* These authors contributed equally to this work.

Full list of GoT2D Consortium members and affiliations

Broad Institute: Jason Flannick, Alisa Manning, Christopher Hartl, Vineeta Agarwala, Pierre Fontanillas, Todd Green, Eric Banks, Mark DePristo, Ryan Poplin, Khalid Shakir, Timothy Fennell, Jacquelyn Murphy, Noël Burtt, Stacey Gabriel, David Altshuler

University of Michigan / FUSION: Christian Fuchsberger, Hyun Min Kang, Xueling Sim, Clement Ma, Adam Locke, Thomas Blackwell, Anne Jackson, Tanya Teslovich, Heather Stringham, Peter Chines, Phoenix Kwan, Jeroen Huyghe, Adrian Tan, Goo Jun, Michael Stitzel, Richard N. Bergman, Lori Bonnycastle, Jaakko Tuomilehto, Francis S. Collins, Laura Scott, Karen Mohlke, Gonçalo Abecasis, Michael Boehnke

Helmholtz München/KORA: Tim Strom, Christian Gieger, Martina Müller-Nurasyid, Harald Grallert, Jennifer Kriebel, Janina Ried, Martin Hrabé de Angelis, Cornelia Huth, Christa Meisinger, Annette Peters, Wolfgang Rathmann, Konstantin Strauch, Thomas Meitinger

Lund University: Jasmina Kravic, Claes Ladenvall, Tiinamaija Toumi, Bo Isomaa, Leif Groop

Univ of Oxford & Wellcome Trust: Kyle Gaulton, Loukas Moutsianas, Manny Rivas, Richard Pearson, Anubha Mahajan, Inga Prokopenko, Ashish Kumar, John Perry, Jeff Chen, Bryan Howie (Chicago), Davis McCarthy, Martijn van de Bunt, Kerrin Small (Kings), Cecilia Lindgren, Gerton Lunter, Neil Robertson, Will Rayner, Andrew Morris, David Buck, Andrew Hattersley (Exeter), Tim Spector (Kings), Gil McVean, Tim Frayling (Exeter), Peter Donnelly, Mark McCarthy

List of all supporting tables and figures

S1 Text: List of all supporting tables and figures; supplemental methods (this file).

S1 Figure: Pairwise linkage disequilibrium between variants in simulated vs. empirical data, for different minor allele frequency categories.

S2 Figure: Comparison of site frequency spectrum at 202 genes in Nelson et al vs. other genes exome-wide.

S3 Figure: Variant frequency-effect size distributions under each simulated architecture.

S4 Figure: Distribution of number of causal variants and total number of simulated variants tested per locus under different architectures.

S5 Figure: Power of gene-based tests under null locus architectures to assess type I error.

S6 Figure: Relative power of gene-based tests using an absolute significance threshold vs. an empirical threshold corrected for the false positive rate of each test.

S7 Figure: Power of gene-based tests in 3K samples, as a function of significance threshold, under each simulated architecture.

S8 Figure: Power of gene-based tests in 3K samples using different minor allele frequency thresholds for burden testing.

S9 Figure: Effect of linkage disequilibrium between causal variants on power of gene-based tests.

S10 Figure: Power of gene-based method (SKAT-O) as compared to single variant association testing under AR4, AR5, and AR6. (Results for AR1, AR2, and AR3 are shown in main manuscript **Fig 3**).

S11 Figure: Power of gene-based tests as a function of locus effect size and sample size.

S12 Figure: Effect of increasing sample size on the simulated number of causal and total segregating variants, and effect of cap on number of causal variants in HAPGEN2.

S13 Figure: Power of gene-based methods in 10K samples, as a function of ratio of causal to total number of variants.

S14 Figure: Concordance between p-values reported by different gene-based association methods under each simulated architecture.

S1 Table: List of human protein-coding loci at which simulations were performed.

S2 Table: Locus architectures modeled at simulated loci.

S3 Table: Power of 'composite' groups of gene-based association methods.

Supplemental description of methods

Generation of simulated reference panels

Simulated datasets were generated using HAPGEN2 (Su et al, 2011). HAPGEN2 generates case-control data using a haplotype reshuffling approach based on the Li & Stephens model. Under this model, simulated (unobserved) haplotypes are assumed to be an imperfect mosaic of actual (observed) haplotypes, and are simulated using a Hidden Markov Model with recombination and mutation rates as parameters. Case-control samples are generated by by over-sampling haplotype segments which contain alleles at which phenotypic effects are introduced (based on the relative risks assigned to them).

In the haplotypes available from the original, 379-strong sample from the 1000 Genomes Project as a reference panel, observed variation is inadequate to simulate effects at rare variants in large samples. We therefore decided to augment this reference panel to a larger final sample size of over 12,000 samples. To do this, we developed a staged, iterative approach using HAPGEN2, by introducing novel variation at each step with the goals of:

i. Maintaining LD structure consistent with what was originally observed in the 1000G panel, *and*
ii. Generating a full site frequency spectrum (SFS) consistent with that observed in empirical re-sequencing data.

Although the mutation rate used as input to HAPGEN2 can be modified to increase the introduction of novel variants, it is based on the Watterson's estimate and assumes a constant population size. Therefore, it cannot be modified to allow for exponential growth in the population, as observed in recent re-sequencing studies (Coventry et al 2010, Keinan et al 2012). In order to obtain a realistic SFS, we followed an approach where (i) an excess of rare variants were introduced using an empirically selected value for the mutation parameter in HAPGEN2, so as to match the SFS for singletons, and (ii) the resulting dataset was subsequently thinned using a rejection sampling approach, in order to match that observed in real re-sequencing data of that size. We used the SFS reported in work by Nelson et al 2012, which was based on sequencing 351 kb of coding sequence in 12,514 samples of European descent as an empirical reference. A value of θ=0.08 was found adequate to generate enough variation to match the singleton count. In order to validate that this approach led to a realistic SFS when sub-sampled to smaller sizes, we compared the SFS of observed in subsets of the simulated, thinned panel with 2,738 individuals to that of empirical exome-wide sequencing data from the GoT2D Project. In both cases, comparisons were performed at protein-coding regions only (main manuscript **Fig 1**).

The building of the augmented simulated panel was conducted in iterative steps, where HAPGEN2 was used to increase the sample size of the simulated reference panel (without introducing phenotypic effects to any variants). Starting from the original set of 379 samples (758 haplotypes), the panel was increased to a final size of 12,514 samples through successive steps of adding 300 individuals (number chosen arbitrarily as a compromise between accuracy and speed; we note that the value of θ=0.08 chosen may need to be altered for different expansion step sizes). At each step, the newly generated simulated samples were added to the reference panel, and the resulting, augmented version was used as reference in the subsequent iteration. The same mutation rate of θ=0.08 was used across all iterations. An illustration of the procedure is shown below.

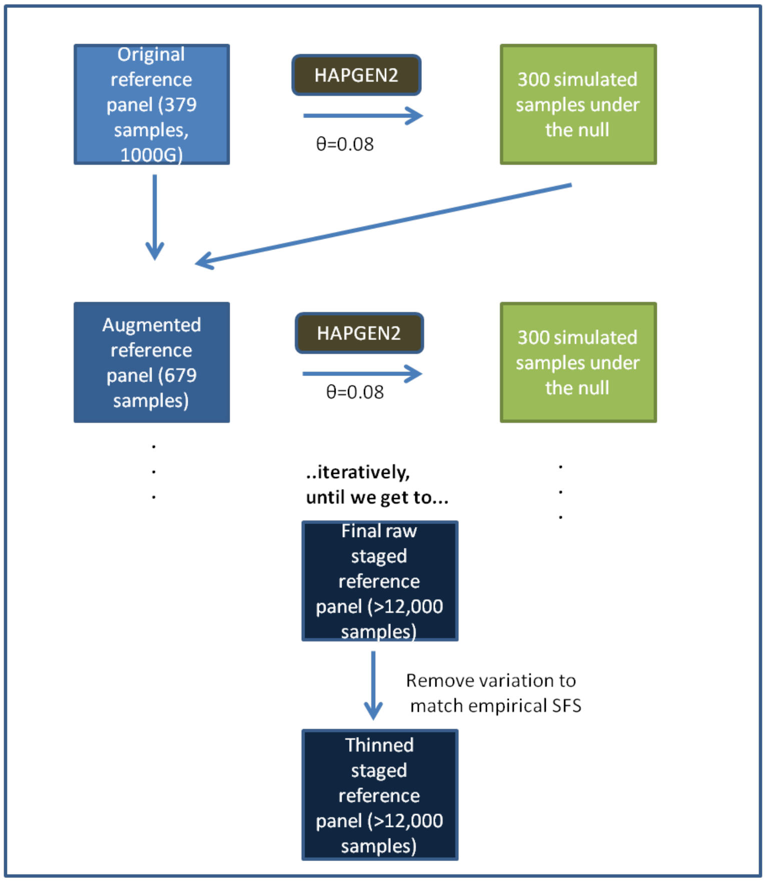

Assignment of phenotypic effects at loci simulated in HAPGEN2

All variant effects were assigned based on the frequency of each allele in the augmented, 12,514-strong reference panel. All deleterious effects were assigned by sampling from frequency-relative risk distributions generated as described by Agarwala et al (*Nature Genetics* 2013) and explained in **S3 Fig**. Protective effects were sampled in the same way, but 50% of the relative risks were inverted. The following procedure was followed for introducing variation at each gene:

i. Pick an exonic variant at random
ii. Introduce an effect by sampling from the frequency-RR distribution for each architecture
iii. If the cumulative variance (on the liability scale) explained by variants at the locus is below the specified threshold, go to step (i) and repeat.
iv. If the variance explained is above the specified threshold, remove one of the introduced variants at random and go to step (i).
v. If for the case of VE = 1%, the variance explained is in (0.0095,0.0105), then
    a. If the number of variants introduced is over 35 – quit and re-start.
    b. Else, accept sampling and simulate data using the variants and effect sizes chosen in HAPGEN2.

The upper threshold of 35 causal variants per locus was a practical limitation, as higher numbers occasionally led to unstable behavior of HAPGEN2. For architectures AR5 and AR6, all exonic variants picked at step (i) had to have an allele frequency of less than or equal to 0.01.

For the calculation of the variance explained by each locus, we employed the method described by So et al. (2011). This approach assumes a multifactorial liability threshold model (Falconer, 1965). According to this model, the overall liability to disease is a continuous function of a number of contributing genetic variants with various effects, as well as of other risk factors. It is assumed to follow a standard normal distribution. For a dichotomous trait, there are two observable states, corresponding to whether an individual will develop the disease or not. The individuals who develop disease are those whose liability exceeds a trait-specific, set threshold. This is determined by the trait's prevalence in the general population, taken here to be 0.08 (but which can be varied by users of the simwrap software provided on the website). To estimate the percentage of genetic variance explained by a single variant, the model takes two additional parameters as input: the frequency of the risk variant in the general population, and the genotype relative risk.

Specifically, let's take A to be the risk allele of a variant at which an effect is introduced, with genotypes aa, Aa and AA. The relative risks for Aa and AA, assuming additivity, are RR1 and RR2=RR1^2, respectively, with $RR1 = \frac{P(D|G=Aa)}{P(D|G=aa)}$. If we assume HWE equilibrium, the frequencies for aa, Aa, and AA are:

$$P(G = aa) = fr(a)^2, P(G = AA) = fr(A)^2 and\ P(G = Aa) = 2 * fr(a) * fr(A)\,.$$

The probability (prevalence) of disease is then

$$K = fr(a)^2 * P(D|G = aa) + 2 * fr(a) * fr(A) * P(D|G = Aa) + fr(A)^2 * P(D|G = AA)),$$

which becomes

$$K = fr(a)^2 * P(D|G = aa) + 2 * fr(a) * fr(A) * RR1 * P(D|G = aa) + fr(A)^2 * RR2 * P(D|G = aa)).$$

Hence, for a given prevalence and relative risk, the above formula can be solved to give the prevalence of genotype aa, $P(D|G = aa)$). Under the liability threshold model, the three genotypes are assumed to have liability distributions with different means but the same residual variance. The genotype-specific mean liabilities are a function of the prevalence of each genotype and the liability threshold and can be estimated by setting one of them to zero.

Assuming independence between the effects of individual risk variants across each locus (i.e. that the covariance between variant genotypes is zero), the total percentage of the variance explained is taken to be the sum of the variance explained by each of the variants with introduced effects. This can easily be shown for two variants A, B at a locus since, if we assume:

$$var(AA + BB) = var(AA) + var(BB) + cov(AA, BB) = var(AA) + var(BB),$$

$$var(AA + Bb) = var(AA) + var(Bb), etc,$$

Then the variance explained by the multi-locus genotype at A, B is (9 genotypic combinations):

$$Vg(A + B) = fr(A)^2 fr(B)^2 \big(var(AA) + var(BB)\big) + 2fr(A)fr(a)fr(B)^2 \big(var(Aa) + var(BB)\big) + fr(a)^2 fr(B)^2 \big(var(aa) + var(BB)\big) + 2fr(B)fr(b)fr(A)^2 \big(var(AA) + var(Bb)\big) + 2fr(B)fr(b)2fr(A)fr(a)\big(var(Aa) + var(Bb)\big) + fr(A)^2 fr(b)^2 \big(var(AA) + var(bb)\big) + 2fr(B)fr(b)fr(a)^2 \big(var(aa) + var(Bb)\big) + 2fr(A)fr(a)fr(b)^2 \big(var(Aa) + var(bb)\big) + fr(a)^2 fr(b)^2 \big(var(aa) + var(bb)\big),$$

which becomes

$Vg(A+B) = fr(A)^2(var(AA)) + 2fr(A)fr(a)(var(Aa)) + fr(a)^2(var(aa)) + fr(B)^2(var(BB)) + 2fr(B)fr(a)(var(Bb)) + fr(b)^2(var(bb)) = Vg(A) + Vg(B)$.

We made the assumption of independence between the effects assigned to each variant at a locus because the vast majority of risk variants under the architectures simulated in this study are rare and the LD between them is minimal. Indeed, the mean pairwise r^2 between risk variants was found to be $r^2>0.05$ for only 62 out of the 2500 datasets simulated under architecture AR2, and $r^2>0.3$ for only 2 of them.

Varying the percentage of neutral variation at a simulated locus

In order to assess how the performance of gene-based tests of association changes with varying degrees of neutral variation at a locus, we followed the following procedure:

- For each dataset, only keep the *C* causal variants which have a frequency of 1% or less.
- Introduce a number (*N*) of neutral variants with a frequency of up to 1%, so that the percentage of causal variants amongst those tested is equal to the desired fraction. For instance, in order to test the power when 50% of the variants tested are causal, the number of neutral variants to be introduced will be N=C. To note:
    i) *C* is *not* the total number of variants with assigned effects across the locus, as variants with AF>1% also have phenotypic effects. Rather this is the number of causal variants included in the burden test.
    ii) The percentage of neutral variation per locus in analyses where all exonic variation with MAF<1% is included (e.g. as shown in **Fig 2, Fig 3**) is variable (typically ~50%). In analyses where the ratio of *C* to *N* is fixed, we allow the total number of variants tested can exceed the total number of exonic variants. This occurs because neutral variants (from across the entire transcript) are included in order to match the required ratio of *C* to *N*. For instance, a dataset with 20 causal variants with an AF≤ 1% and 30 exonic variants in total will have 30 variants tested in **Fig 2, Fig 3**, whereas when we fix 50% of the variants be causal, 20 neutral variants will be included instead, raising the total number of variants tested to 40. In this scenario, power is reduced.
    iii) Datasets in which the total number of neutral variants present across the entire transcript is not enough to introduce the required percentage of *C* to *N* are excluded from the power estimation.

Selecting a subset of tests for joint application to the data

The subsets of tests chosen for inclusion into the composite test were picked using a stepwise forward selection approach. Starting from a single test, the next test to be included at each round was the one which offered the highest number of novel hits amongst all remaining loci. Novel hits are defined as datasets for which the p-value of the new test to be added was lower by at least a given margin when compared to the lowest p-value of the tests already included in the composite test. Three margins were used (p_value_composite below indicates the lowest p-value reported by all tests included in the composite test at a given stage of selection):

i. 100, which indicates a (fold) difference of at least two orders of magnitude (p_value_new<100*p_value_composite),
ii. 10, which indicates a (fold) difference of at least an order of magnitude p_value_new<10*p_value_composite), and

iii. 1, which indicates any difference between the test to be added and the composite test (such that p_value_new<p_value_composite).

A higher margin reflects a greater ability of the test to be added to increase the number of "truly novel" hits (hits uniquely detected by the test to be added); a margin of 1, on the other hand, means that the test to be added at each step is the one which offers the highest absolute increase in power, regardless of the degree of uniqueness. Results for all architectures, starting from KBAC, MiST or SKAT-O, are shown in **Table S15**. The tests selected to comprise the composite one are shown in the order that they were picked by the above described algorithm.

We note that this approach takes into account correlation between the test statistics reported by each method. The reported power (sensitivity) and false positive rate are based on empirical results of each method on the simulated datasets. The tests were run on datasets simulated both under the architectures described in the manuscript as well as under the null (where no variants were assigned phenotypic effects).

- After the inclusion of each additional test in the composite one, the sensitivity of the latter was increased only if the addition had led to an increased number of true positives. Hence, if the results of the additional test on the datasets simulated under the alternative were perfectly correlated with a test already included in the composite, no change in sensitivity would occur.
- Conversely, an increase in the number of false positives would only occur if the additional test showed significant association with a null dataset for which all other tests already included in the composite test had shown no association. In that case, the false positive rate would increase and an adjustment would be required to maintain correct behavior under the null. This was done by adjusting (lowering) the p-value threshold so that the FDR for the new composite test would remain less than 1*10^-4.

If the p-value threshold for significance is kept at α=1e-04, the power (sensitivity) of the composite test will increase with each new addition until all tests are included, or until no other test detects novel signals. This occurs at the expense of an increased false positive rate (FPR). The power of the composite test and the respective FPR (calculated using 100K null simulations) are shown in **S3 Table**. When the p-value is adjusted at each iteration in order to keep the composite test well-calibrated (that is, with a false positive rate of 1e-04), we find that the power of the composite test does not necessarily increase after each step. The power of the composite test under the adjusted p-value threshold is also shown in **S3 Table**.

The application of Fisher's method (not corrected for dependence between tests) as the test-statistic, instead of just using the lowest p-value amongst all tests in the composite one, resulted in lower sensitivity of the composite test.
