# Supplementary material for: The Power of Gene-Based Rare Variant Methods to Detect Disease-Associated Variation and Test Hypotheses About Complex Disease
Source: PLoS Genet. 2015 Apr 23;11(4):e1005165. doi: 10.1371/journal.pgen.1005165 (PMC4407972; doi:10.1371/journal.pgen.1005165)

S1 Figure: Pairwise linkage disequilibrium between variants in simulated vs. empirical data, for different minor allele frequency categories.

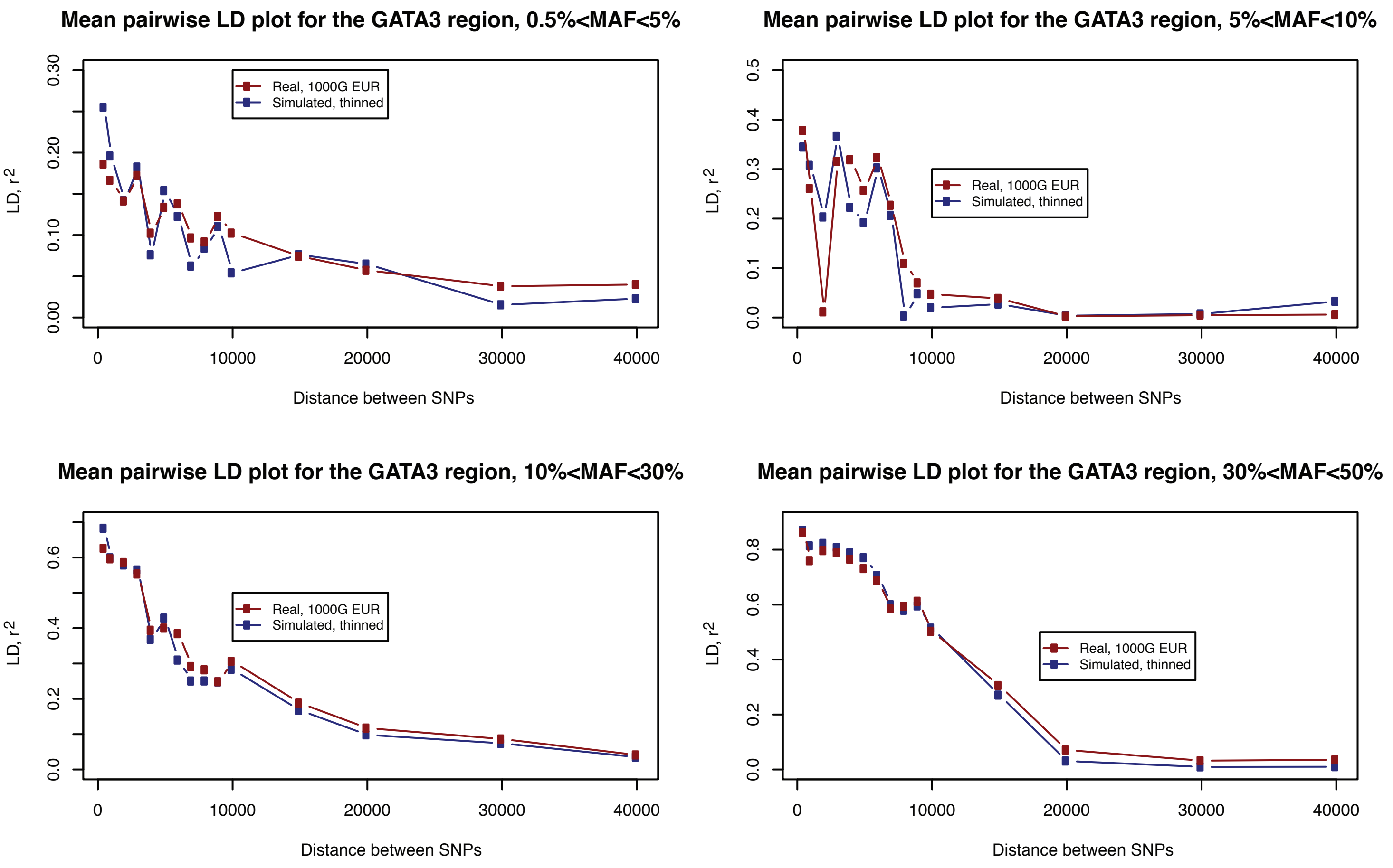

Supplement: S1 Fig — Mean pairwise LD between variants (measured by r2) as a function of the distance between each pair of variants. The pairwise r is shown for the empirical 1000 Genomes reference panel used in this study (red lines) and variants in the simulated panel which was expanded using HAPGEN2 (blue lines; see S1 Text for details about expansion). Each plot shows variants split by minor allele frequency category; in all cases, linkage in the simulated data mimic what is seen in empirical data. Data is shown at a single representative gene locus (GATA3). The mean pairwise LD was calculated and averaged across 10 different subsets (each containing 379 samples, to match the size of the 1000 Genomes European reference panel) of data simulated using HAPGEN2 at the GATA3 locus. (PDF) [file pgen.1005165.s002.pdf]
