# Supplementary material for: The Power of Gene-Based Rare Variant Methods to Detect Disease-Associated Variation and Test Hypotheses About Complex Disease
Source: PLoS Genet. 2015 Apr 23;11(4):e1005165. doi: 10.1371/journal.pgen.1005165 (PMC4407972; doi:10.1371/journal.pgen.1005165)

S2 Figure: Comparison of site frequency spectrum at 202 Nelson et al genes vs. all other REFSEQ genes.

Comparison of the full site frequency spectrum (all MAF) at Nelson et al genes vs. other REFSEQ genes:

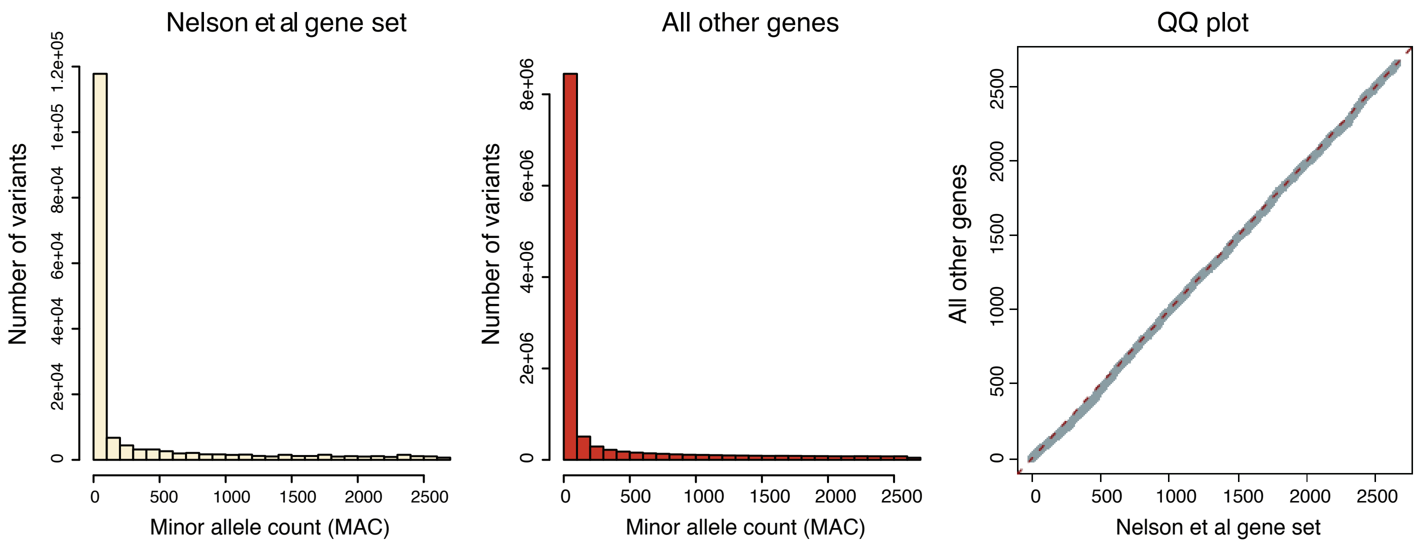

Comparison of low frequency SFS (MAF<1% only) at Nelson et al genes vs. other REFSEQ genes:

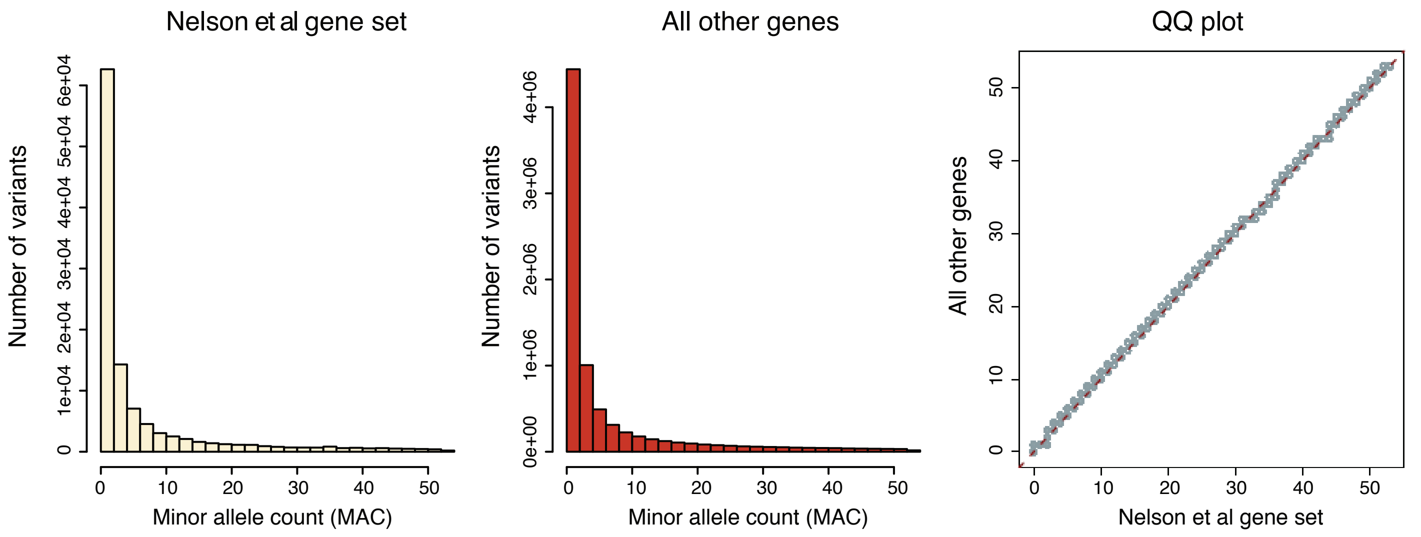

Supplement: S2 Fig — All simulations discussed in this manuscript were conducted (in HAPGEN2) to match the empirical site frequency spectrum reported across 202 genes in Nelson et al (Science, 2012; main text reference 14). This comparator dataset was chosen because of its large sample size (12K individuals). To confirm that the site frequency spectrum across these 202 genes (‘Nelson et al gene set’) is representative of the distribution across all genes, we compared the SFS at these genes to that observed for all other genes in REFSEQ across the genome (‘All other genes’) in a dataset of 2,657 European individuals who were whole-exome sequenced by the Go-T2D Consortium. These data confirm that the Nelson et al genes are not outliers on the basis of observed minor allele counts. (PDF) [file pgen.1005165.s003.pdf]
