# Supplementary figures and images for: The Power of Gene-Based Rare Variant Methods to Detect Disease-Associated Variation and Test Hypotheses About Complex Disease
Source: PLoS Genet. 2015 Apr 23;11(4):e1005165. doi: 10.1371/journal.pgen.1005165 (PMC4407972; doi:10.1371/journal.pgen.1005165)

**S3 Figure: Variant frequency-effect size distributions under each simulated architecture.**

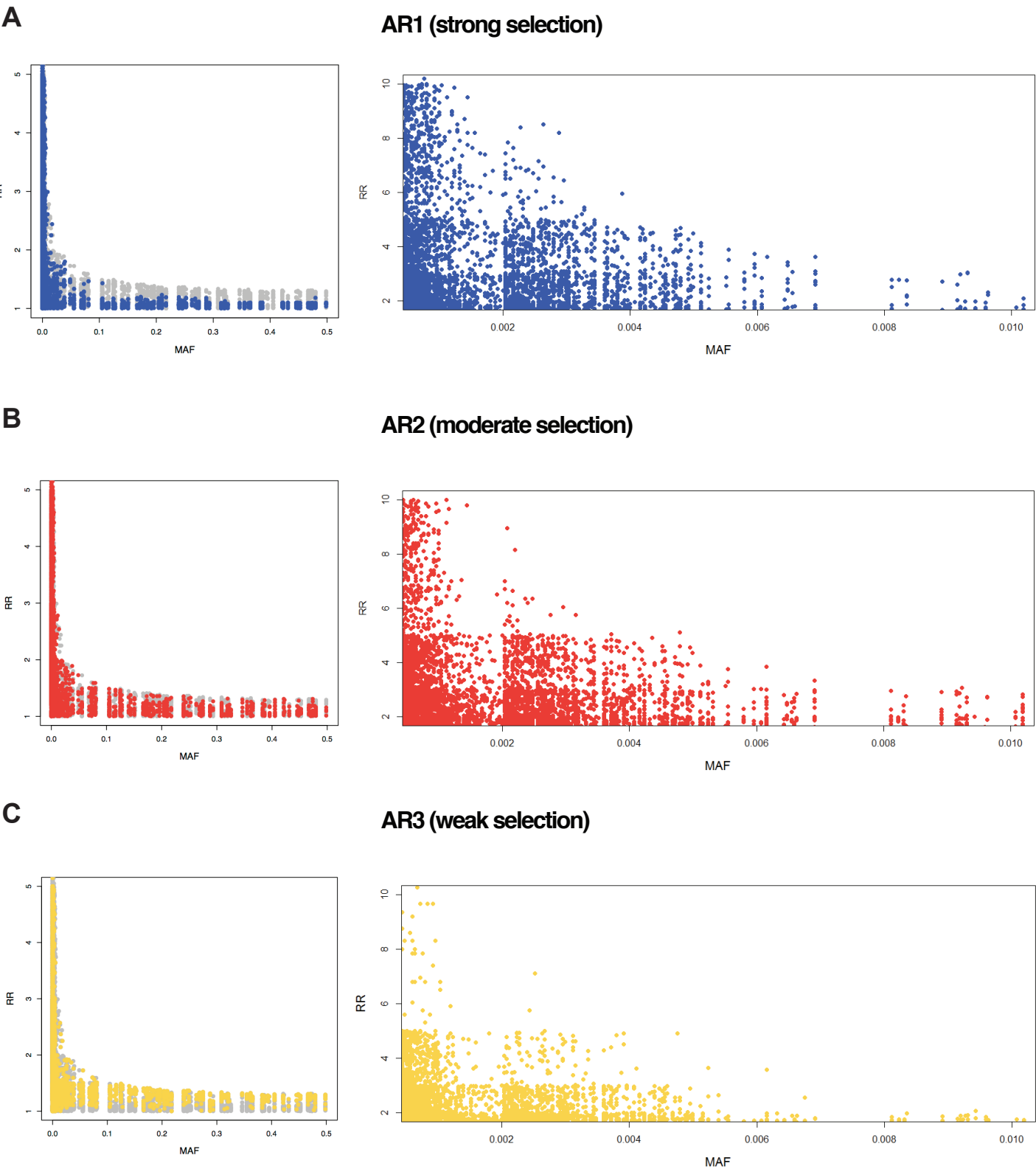

Supplement: S3 Fig — The below frequency-RR distributions were learned from population genetic forward simulations of global genetic architecture (Agarwala et al, Nature Genetics 2013). (A) AR1 assumes strong coupling to purifying selection; that is, variants under selection (more likely rare) have larger effects on disease. (B) AR2 assumes moderate coupling to selection, and (C) AR3 assumes no coupling to selection (thus effect sizes are more uniform across the frequency spectrum). Figures on the right are zoomed-in versions of those on the left (only showing variants with MAF up to 1%). (PDF) [file pgen.1005165.s004.pdf]

S5 Figure: Power of gene-based under null locus architectures to assess type I error.

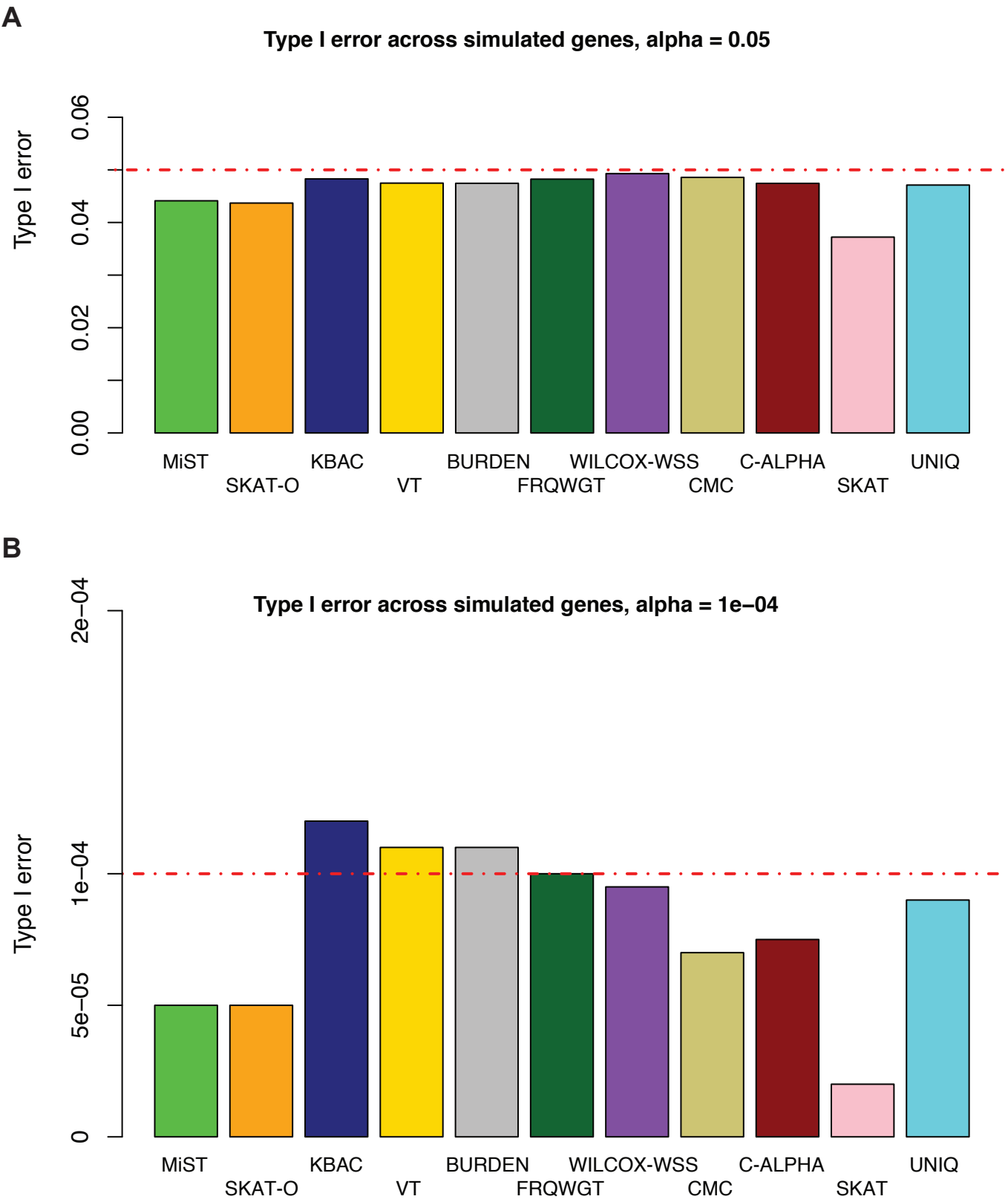

Supplement: S5 Fig — All gene-based tests were relatively well-calibrated and had expected type I error rates at both (A) alpha = 0.05 and (B) alpha = 1e-04. Some tests, such as SKAT, appear to be relatively conservative (as has been previously described). (PDF) [file pgen.1005165.s006.pdf]

S11 Figure: Power of gene-based tests as a function of locus effect size and sample size.

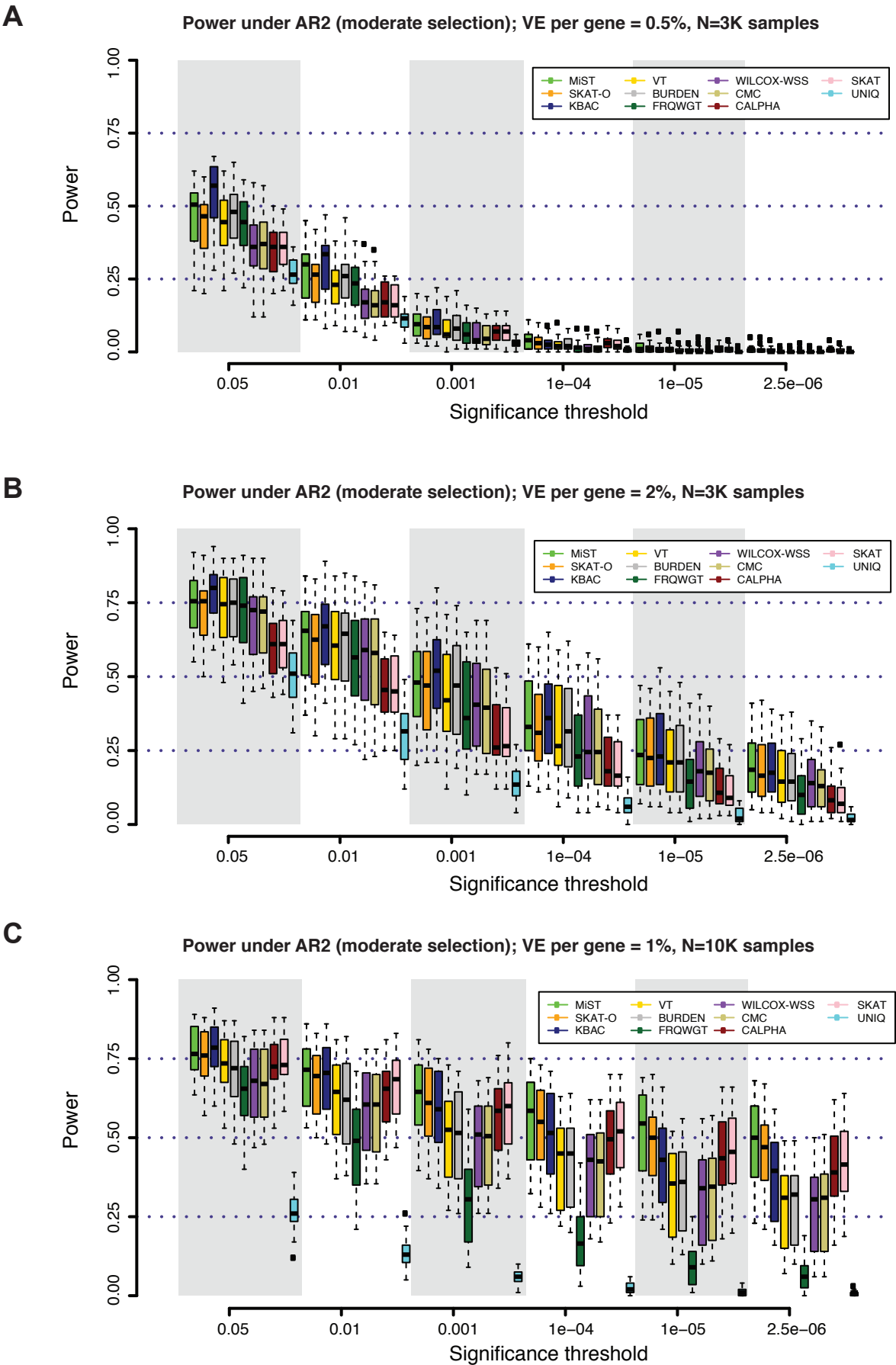

Supplement: S11 Fig — Power is shown here under AR2 (moderate coupling to selection) for varying locus effect sizes and sample sizes. (A) VE = 0.5%, per locus, N = 3K samples, (B) VE = 2%, N = 3K samples, (C) VE = 1%, 10K samples. In Fig 2 of the main manuscript, data was shown for VE = 1% and N = 3K individuals, across a range of architectures. (PDF) [file pgen.1005165.s012.pdf]
