# Supplementary material for: The Power of Gene-Based Rare Variant Methods to Detect Disease-Associated Variation and Test Hypotheses About Complex Disease
Source: PLoS Genet. 2015 Apr 23;11(4):e1005165. doi: 10.1371/journal.pgen.1005165 (PMC4407972; doi:10.1371/journal.pgen.1005165)

**S4 Figure: Distribution of number of causal variants and total number of simulated variants tested per locus under different architectures.**

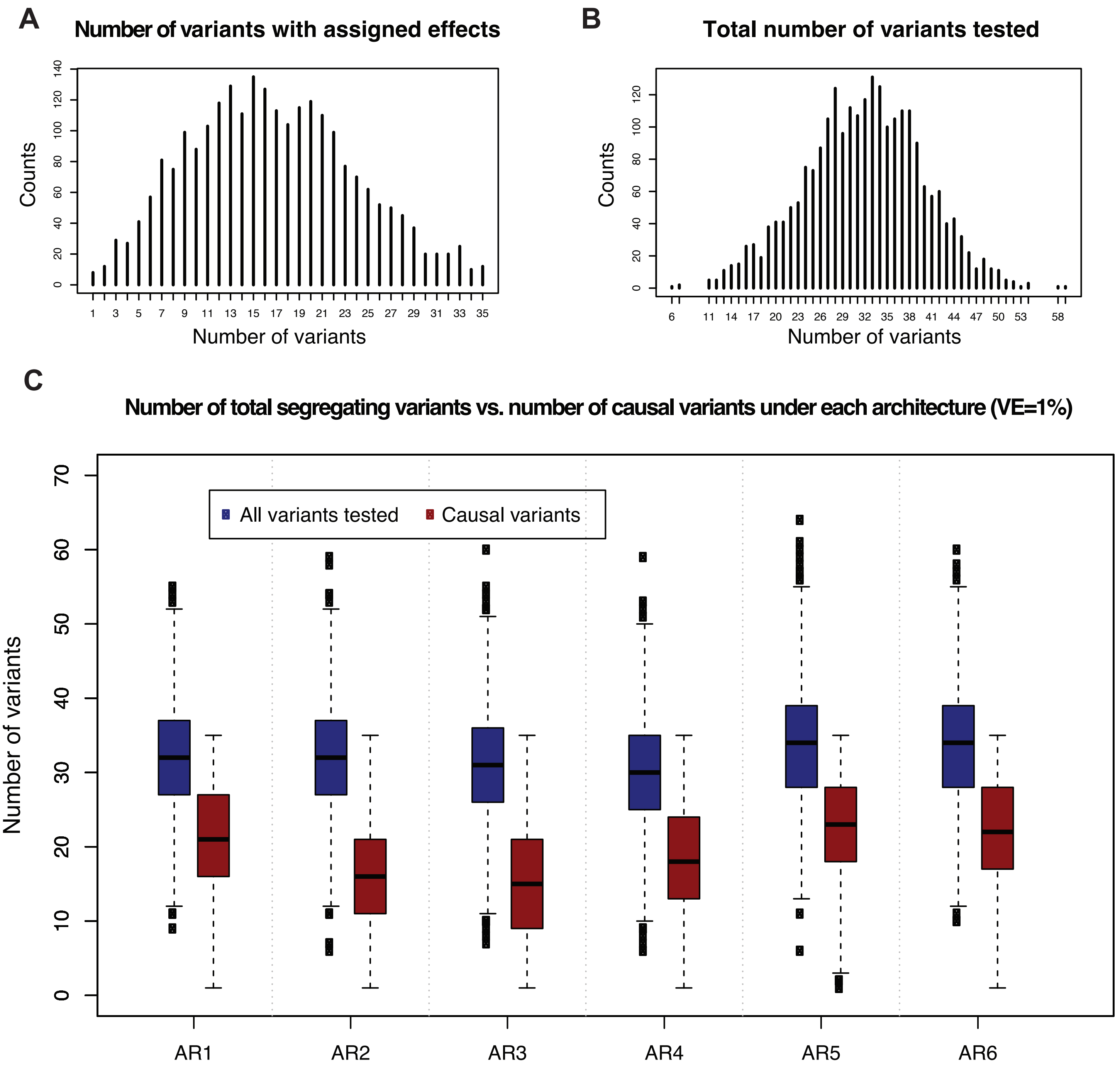

Supplement: S4 Fig — All results shown below are for loci which explain 1% of phenotypic variance, simulated in 3K samples (1.5 cases / 1.5K controls). In (A) and (B) variant counts are shown for the simulated architecture AR2 (moderate selection). In (A) is shown a histogram of simulated loci, binned by the number of causal variants sampled per locus in order for the locus to explain 1% of phenotypic variance. In (B) is shown a histogram of simulated loci, binned now by the total number of exonic variants with MAF<1%, e.g. the total number of variants included in gene-based association testing. In (C), distributions of variant counts (for both causal variants and the total number of variants tested per locus) are shown as box plots, under all six simulated architectures (S2 Table). (PDF) [file pgen.1005165.s005.pdf]
