# Supplementary material for: The Power of Gene-Based Rare Variant Methods to Detect Disease-Associated Variation and Test Hypotheses About Complex Disease
Source: PLoS Genet. 2015 Apr 23;11(4):e1005165. doi: 10.1371/journal.pgen.1005165 (PMC4407972; doi:10.1371/journal.pgen.1005165)

S6 Figure: Relative power of gene-based tests using an absolute significance threshold vs. an empirical threshold corrected for the false positive rate of each test.

A

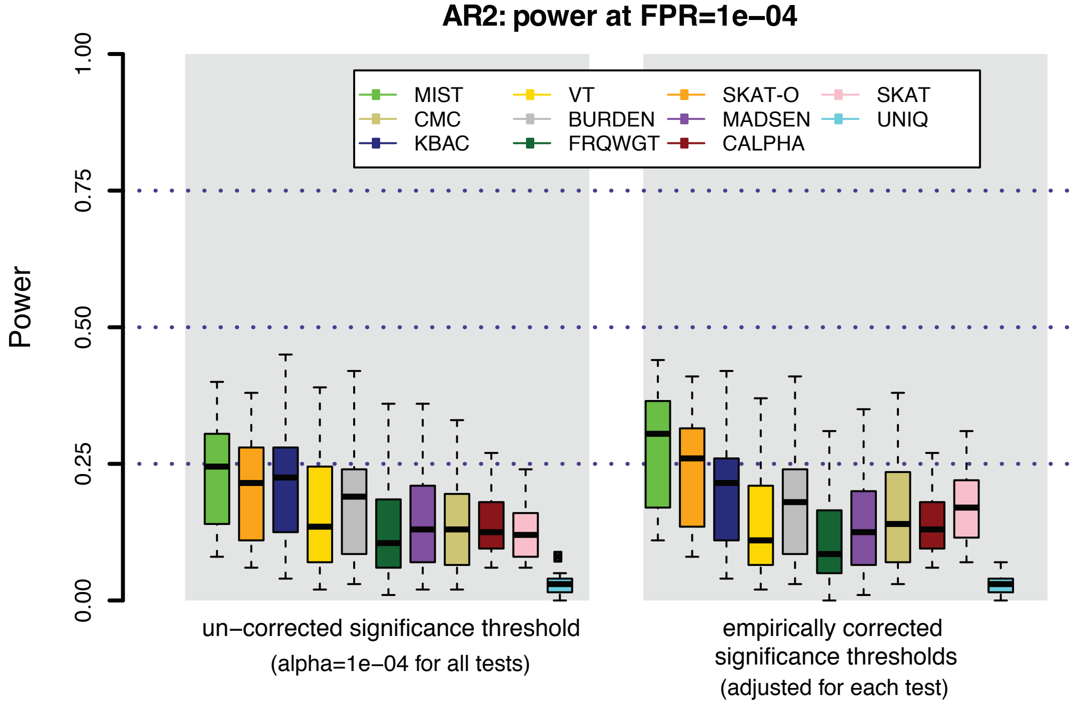

B

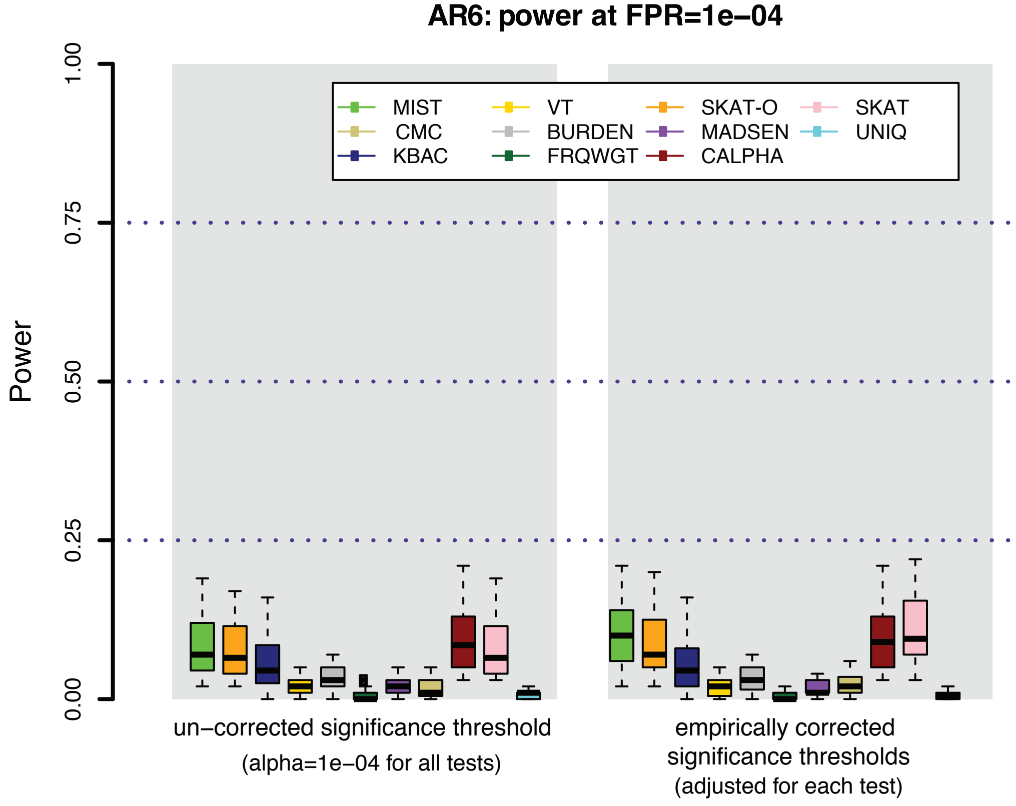

Supplement: S6 Fig — All results shown below are for loci which explain 1% of phenotypic variance, simulated in 3K samples (1.5 cases / 1.5K controls). Results in (A) were simulated under architecture AR2 (moderate selection, unidirectional effects); results in (B) were simulated under AR6 (moderate selection, bidirectional effects). FPR-corrected power is calculated using an empirically-derived threshold at which the observed false positive rate is 1e-04; this threshold varies for each gene-based method based on how conservative each method is (this variability is shown in S8 Fig). The relative power of the highest-ranked methods is unchanged with corrected vs. uncorrected significance thresholds. (PDF) [file pgen.1005165.s007.pdf]
