# Supplementary material for: The Power of Gene-Based Rare Variant Methods to Detect Disease-Associated Variation and Test Hypotheses About Complex Disease
Source: PLoS Genet. 2015 Apr 23;11(4):e1005165. doi: 10.1371/journal.pgen.1005165 (PMC4407972; doi:10.1371/journal.pgen.1005165)

S7 Figure: Power of gene-based tests in 3K samples, as a function of significance threshold, under each simulated architecture.

A

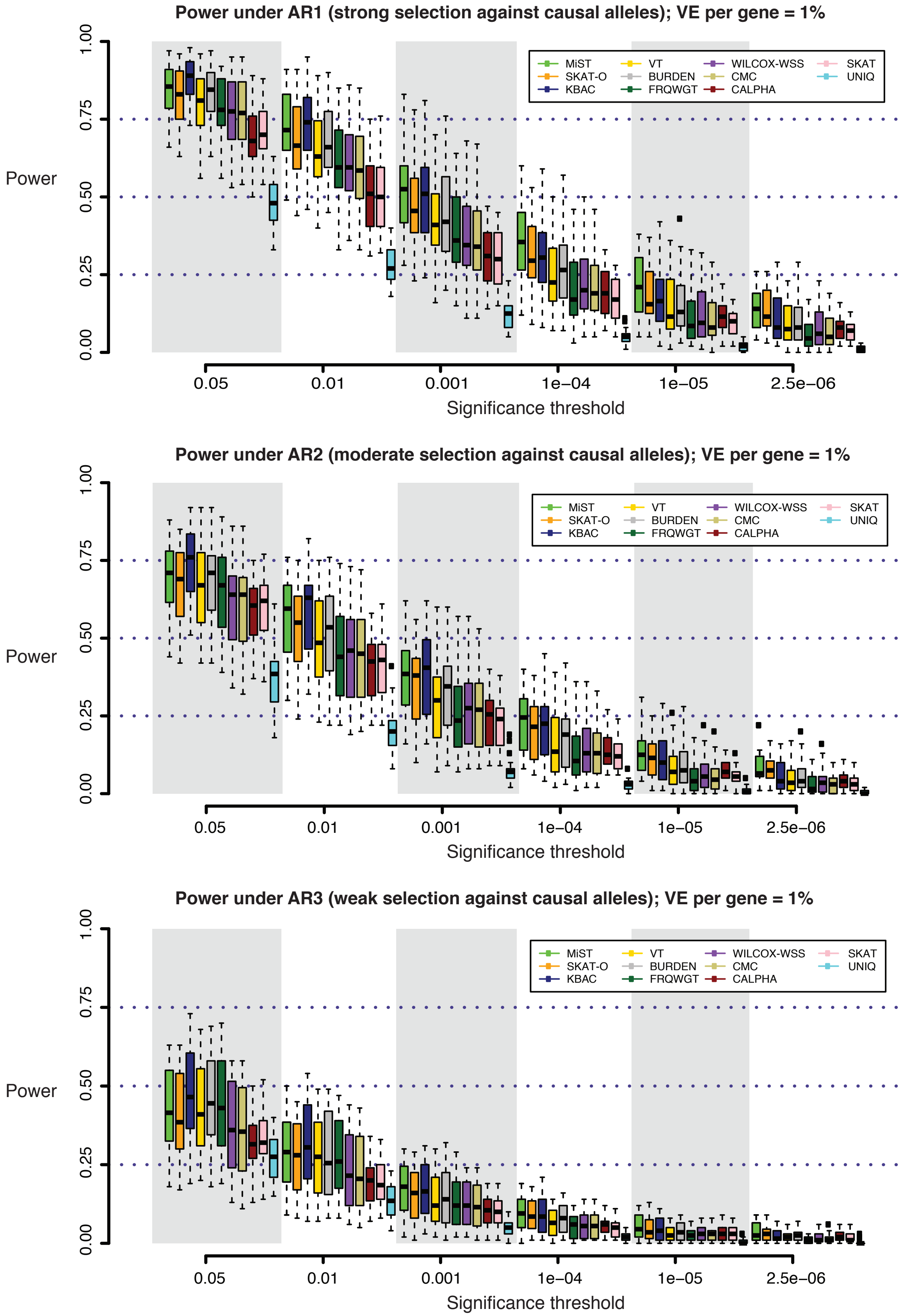

S7 Figure: Power of gene-based tests in 3K samples, as a function of significance threshold, under each simulated architecture (continued).

B

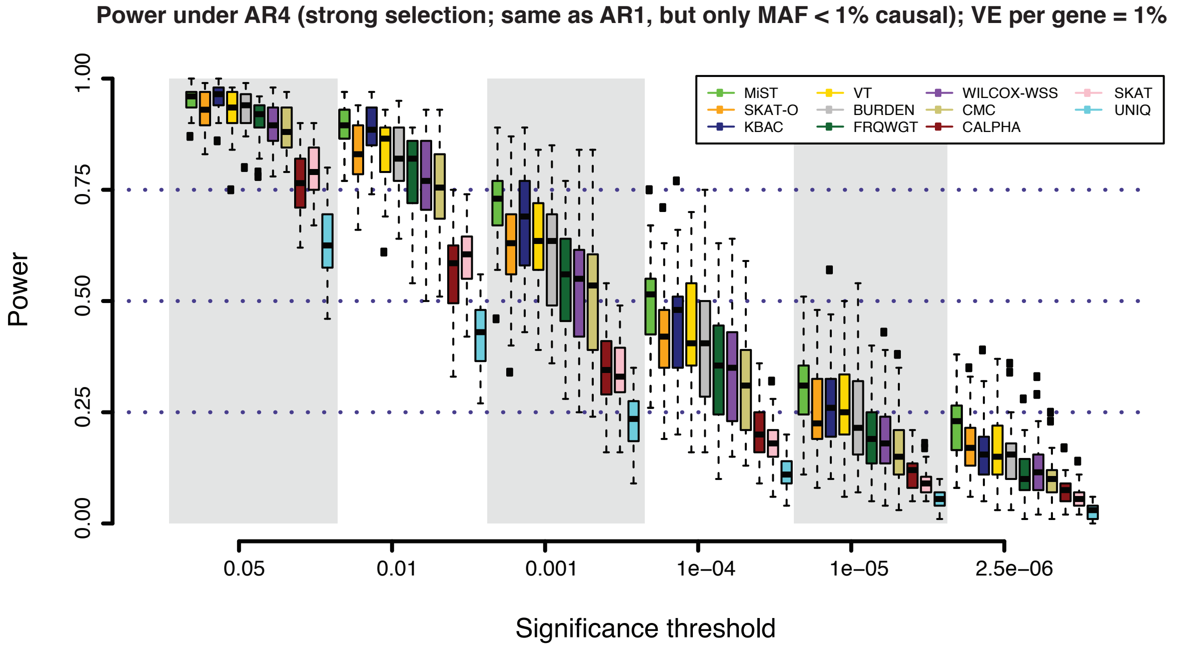

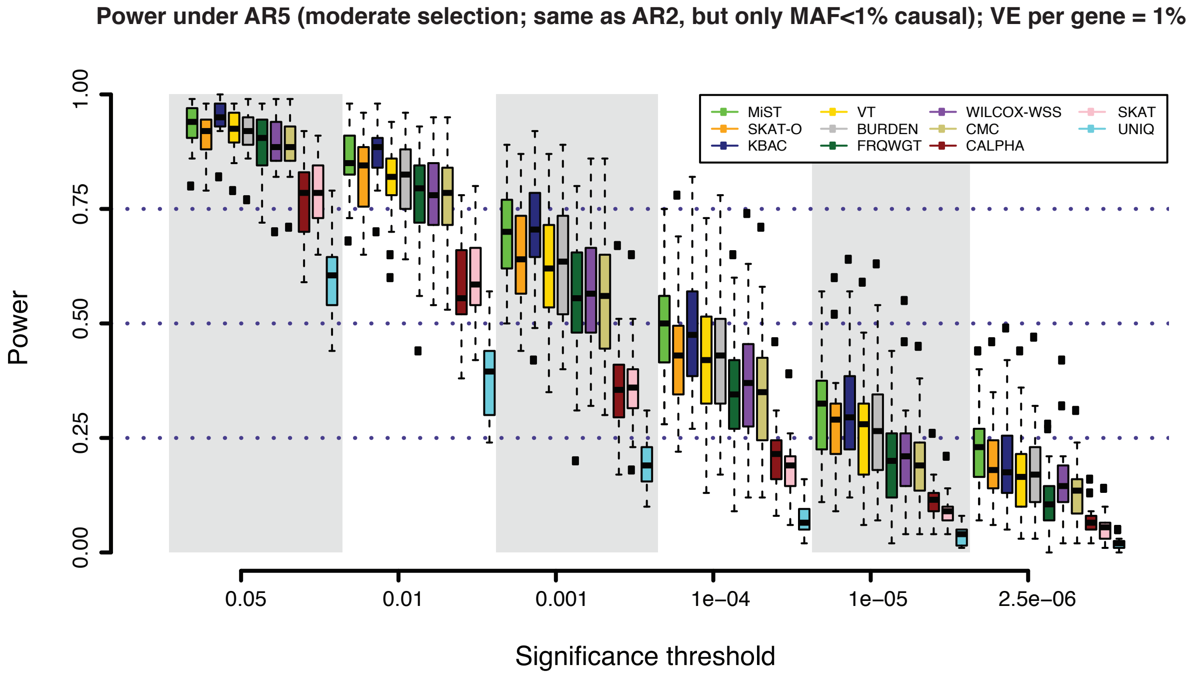

S7 Figure: Power of gene-based tests in 3K samples, as a function of significance threshold, under each simulated architecture (continued).

C

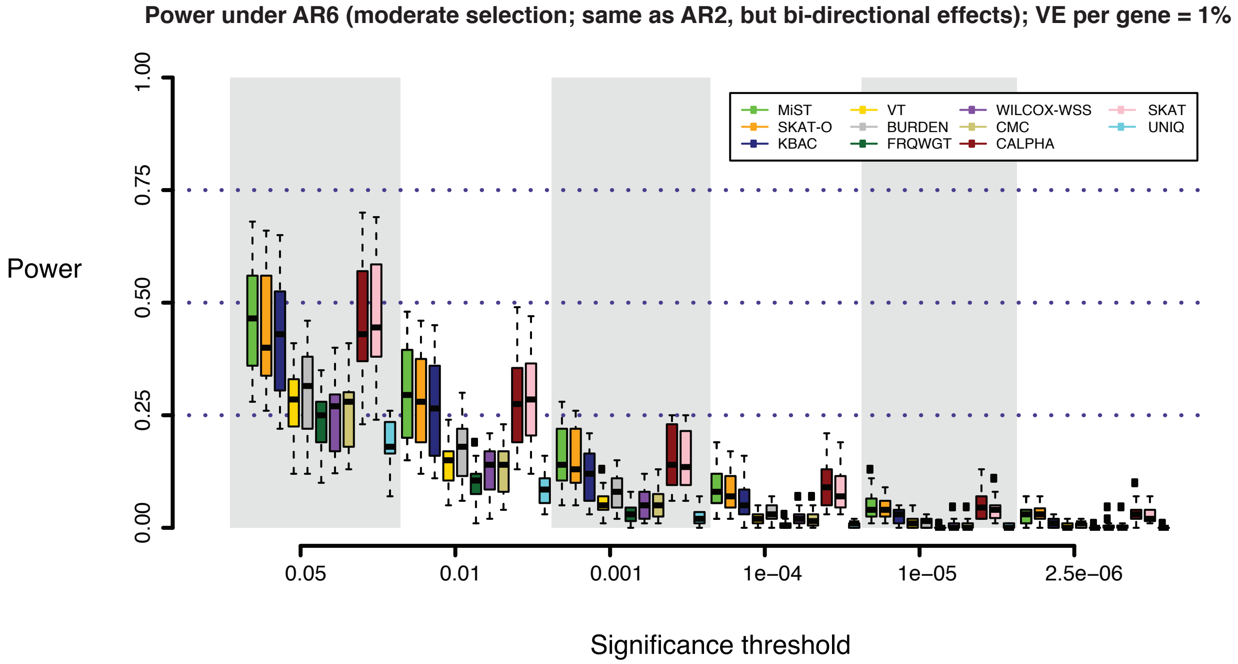

Supplement: S7 Fig — Causal variants at each simulated gene explain 1% of phenotypic variance. In (A), architectures were simulated with causal variants spanning the full frequency spectrum (including common alleles), and all causal alleles increase risk of disease. Gene-based association testing was performed only on variants with MAF < 1%. In (B), only variants with MAF<1% are causal (thus rare alleles are responsible for the entire locus contribution to heritability). In (C), variants across the full site frequency spectrum are causal, but causal alleles are mixed in direction of effect; some increase risk of disease, while others reduce risk of disease. (PDF) [file pgen.1005165.s008.pdf]
