# Supplementary material for: The Power of Gene-Based Rare Variant Methods to Detect Disease-Associated Variation and Test Hypotheses About Complex Disease
Source: PLoS Genet. 2015 Apr 23;11(4):e1005165. doi: 10.1371/journal.pgen.1005165 (PMC4407972; doi:10.1371/journal.pgen.1005165)

S8 Figure: Power of gene-based methods in 3K samples using different minor allele frequency thresholds for burden testing.

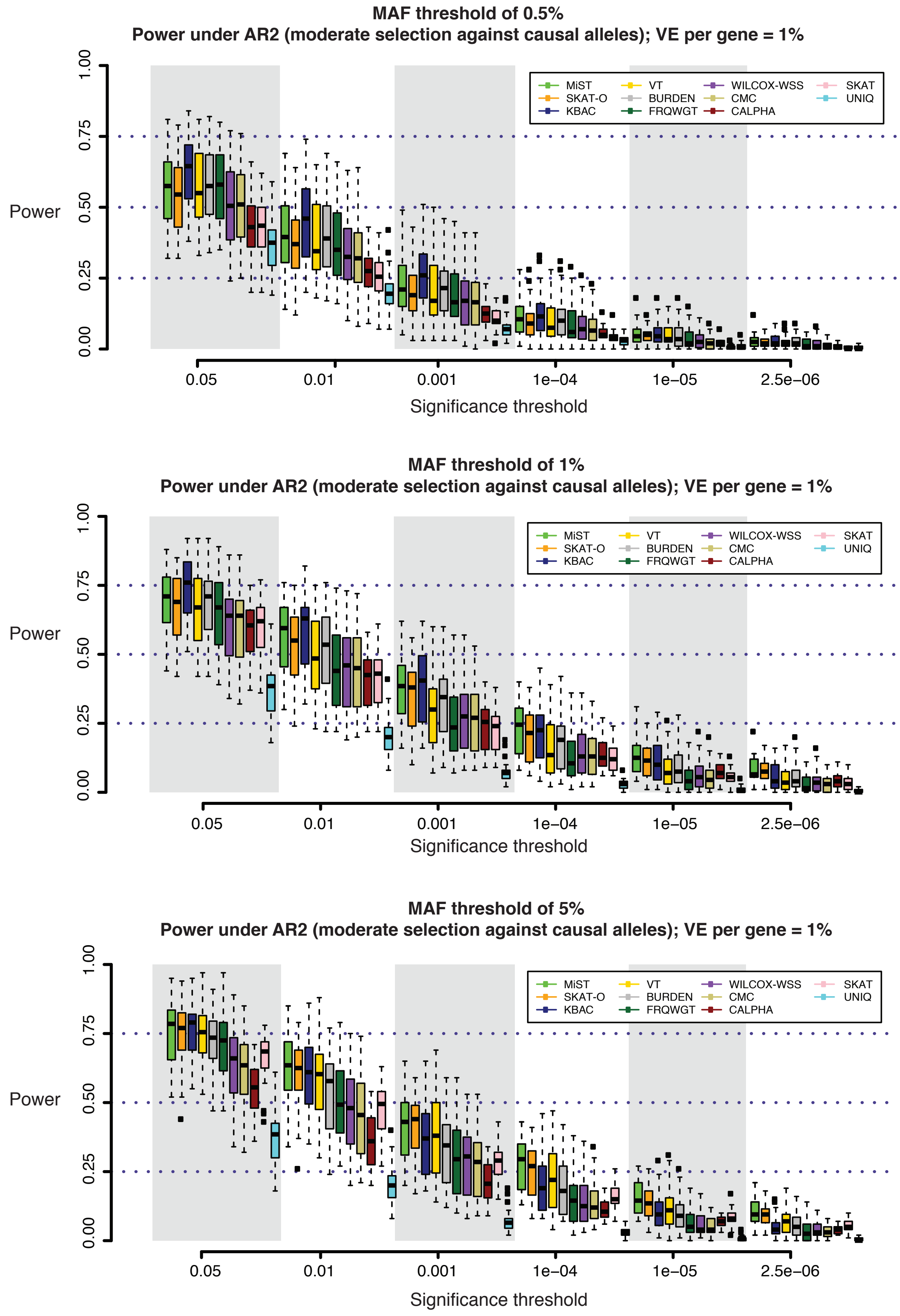

Supplement: S8 Fig — Causal variants at each simulated gene explain 1% of phenotypic variance (contributed by variants across the full frequency spectrum). In Figs 2 and 3 of the main manuscript, a MAF threshold of 1% is used for inclusion of variants into the gene-based association test. Shown here are power results using a MAF threshold of 0.5% and 1%. All simulations below were conducted under AR2 (moderate selection against causal alleles), for loci explaining 1% of phenotypic variance, and in 3K samples. (PDF) [file pgen.1005165.s009.pdf]
