# Supplementary material for: The Power of Gene-Based Rare Variant Methods to Detect Disease-Associated Variation and Test Hypotheses About Complex Disease
Source: PLoS Genet. 2015 Apr 23;11(4):e1005165. doi: 10.1371/journal.pgen.1005165 (PMC4407972; doi:10.1371/journal.pgen.1005165)

S9 Figure: Effect of linkage disequilibrium between causal variants on power of gene-based tests.

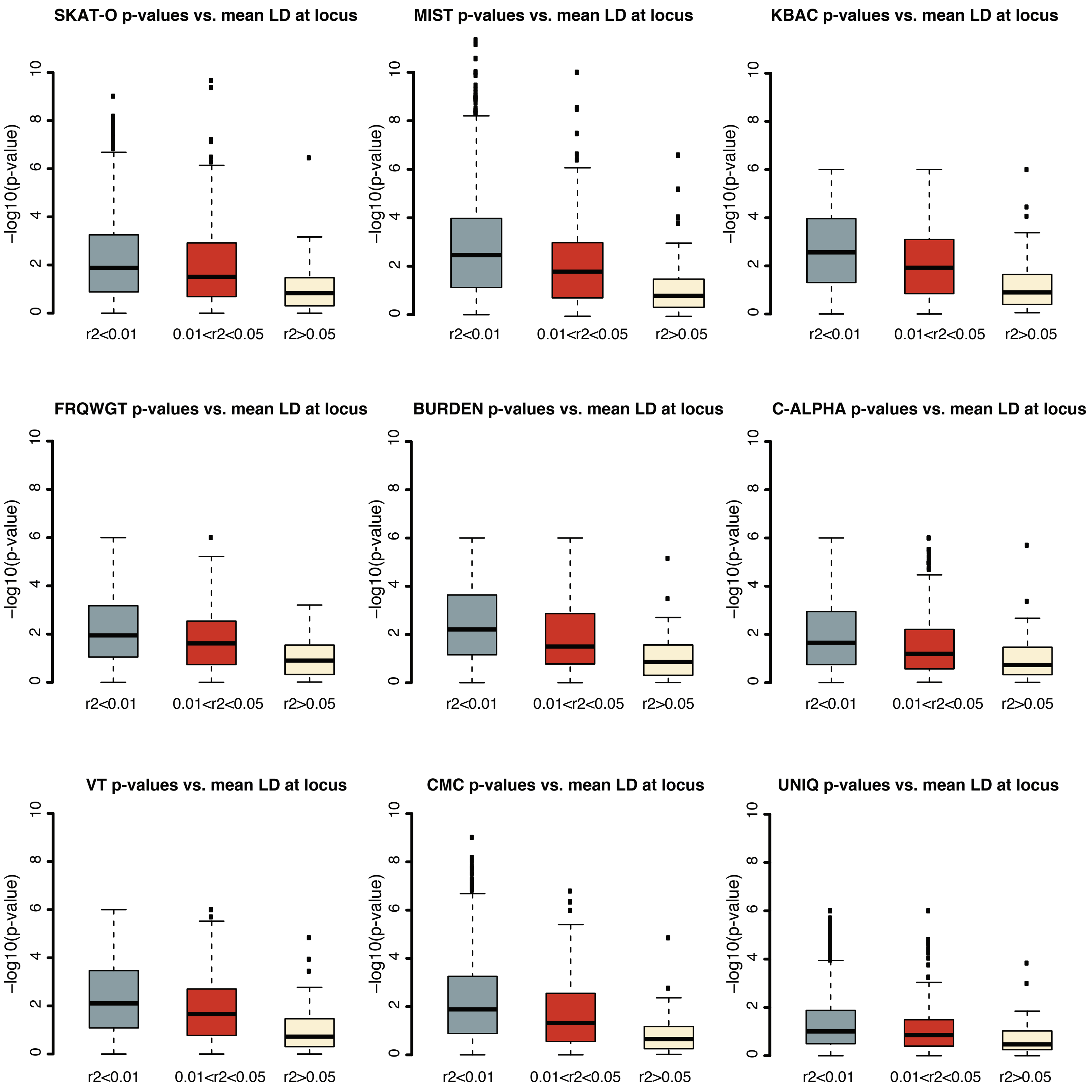

Supplement: S9 Fig — Each boxplot below shows the range of p-values reported by each gene-based method for loci binned by the average pairwise linkage disequilibrium (LD; as measured by r2). Across all the gene-based tests evaluated here, the mean p-values are lower (i.e., more significant) when the extent of LD between causal variants at the locus is low. (PDF) [file pgen.1005165.s010.pdf]
