# Supplementary material for: The Power of Gene-Based Rare Variant Methods to Detect Disease-Associated Variation and Test Hypotheses About Complex Disease
Source: PLoS Genet. 2015 Apr 23;11(4):e1005165. doi: 10.1371/journal.pgen.1005165 (PMC4407972; doi:10.1371/journal.pgen.1005165)

S10 Figure: Power of gene-based method (SKAT-O), as compared to single variant association testing under AR4, AR5, and AR6.

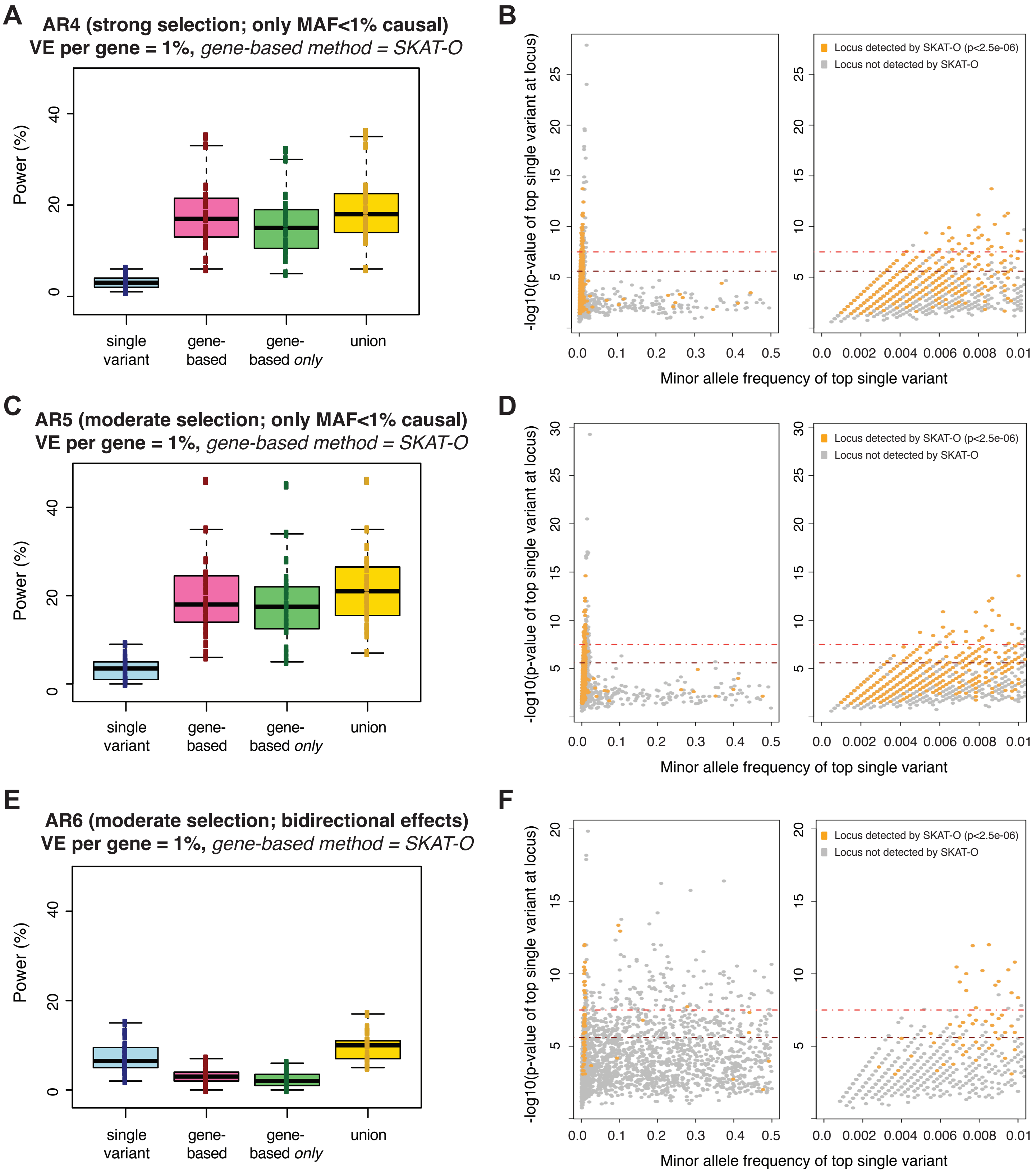

Supplement: S10 Fig — Power is measured across one hundred simulations of phenotypic effects at each of 24 human gene loci in N = 3K samples. Under each architecture (AR4, AR5, AR6), the power of one of the best-performing gene-based tests (SKAT-O) at alpha = 2.5e-06 is compared to single variant association (A,C,E). The significance threshold used for the gene-based test is 2.5e-06; the threshold for single variant association (Fisher’s exact) is 5e-08. Blue boxplot shows range of power for single variant association across genes simulated; pink shows power of the gene-based test; green shows the fraction of loci detected only by the gene-based test (and not single variant association); yellow shows the combined sensitivity of both gene-based and single variant association. Next to each boxplot (panels B,D,F) are scatterplots showing the distinct sets of loci detected by single variant association (loci above the upper dotted red line at 5e-08) and by gene-based association (highlighted in orange). Loci are plotted based on the minor allele frequency (x-axis) and association p-value (y-axis) of the most associated single variant across the locus. Similar plots for AR1, AR2, and AR3 are shown in Fig 3 of the main manuscript. (PDF) [file pgen.1005165.s011.pdf]
