# Supplementary material for: The Power of Gene-Based Rare Variant Methods to Detect Disease-Associated Variation and Test Hypotheses About Complex Disease
Source: PLoS Genet. 2015 Apr 23;11(4):e1005165. doi: 10.1371/journal.pgen.1005165 (PMC4407972; doi:10.1371/journal.pgen.1005165)

S12 Figure: Effect of increasing sample size on the simulated number of causal and total segregating variants per locus, and effect of cap on number of causal variants in HAPGEN2.

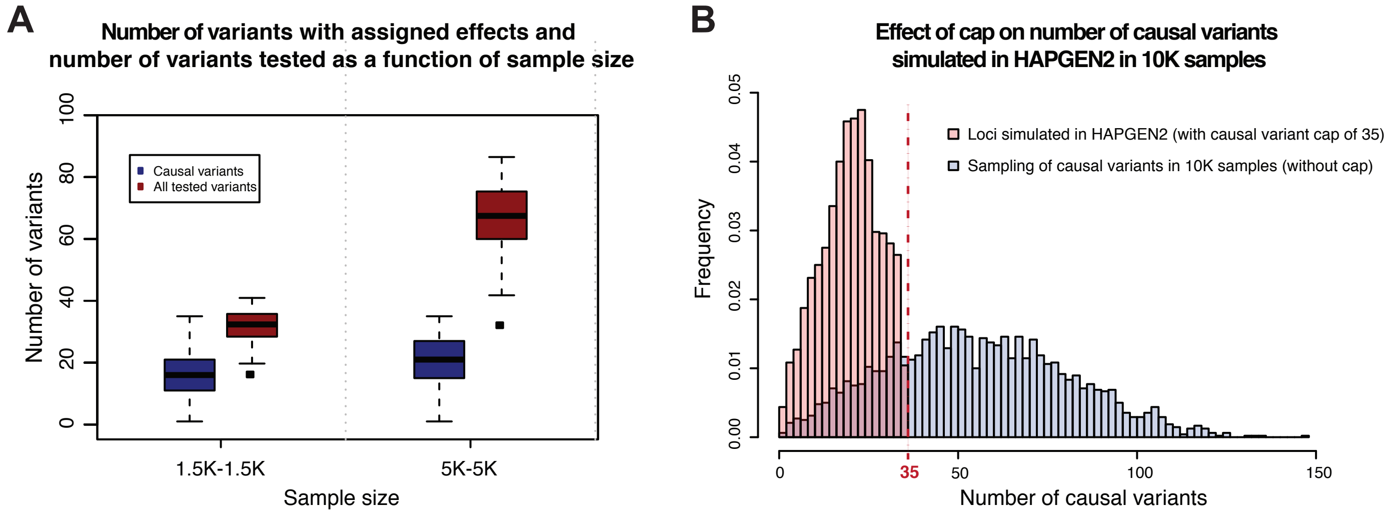

Supplement: S12 Fig — We simulated loci at which the site frequency spectrum (and thus the total number of segregating variants) matched empirical datasets (see main manuscript Fig 1). At these loci, causal variants were sampled from different frequency-effect size distributions; due to technical limitations of HAPGEN2, a maximum of 35 causal variants were selected to collectively explain 1% of phenotypic variance. As this figure demonstrates, this cap did not materially impact simulations in 3K samples, but in 10K samples, this variant cap does restrict the diversity of locus architectures that we were able to simulate. In 3K samples, the median total number of segregating variants with MAF<1% per locus is ~38; ~18 of these variants (well below 35) have causal effects on disease. In 10K samples, the ratio of causal to total variants at simulated loci is substantially reduced due to the cap on the number of causal variants. (A) The number of total segregating variants (red) and the number of variants simulated to have causal effects (blue) per locus. (B) The distribution of number of causal variants per locus in 10K samples with and without the variant cap. We only simulated loci with fewer than 35 causal variants (pink distribution). (PDF) [file pgen.1005165.s013.pdf]
