# Supplementary material for: The Power of Gene-Based Rare Variant Methods to Detect Disease-Associated Variation and Test Hypotheses About Complex Disease
Source: PLoS Genet. 2015 Apr 23;11(4):e1005165. doi: 10.1371/journal.pgen.1005165 (PMC4407972; doi:10.1371/journal.pgen.1005165)

S13 Figure: Power of gene-based methods in 3K vs. 10K samples, as a function of ratio of causal to total number of variants per locus.

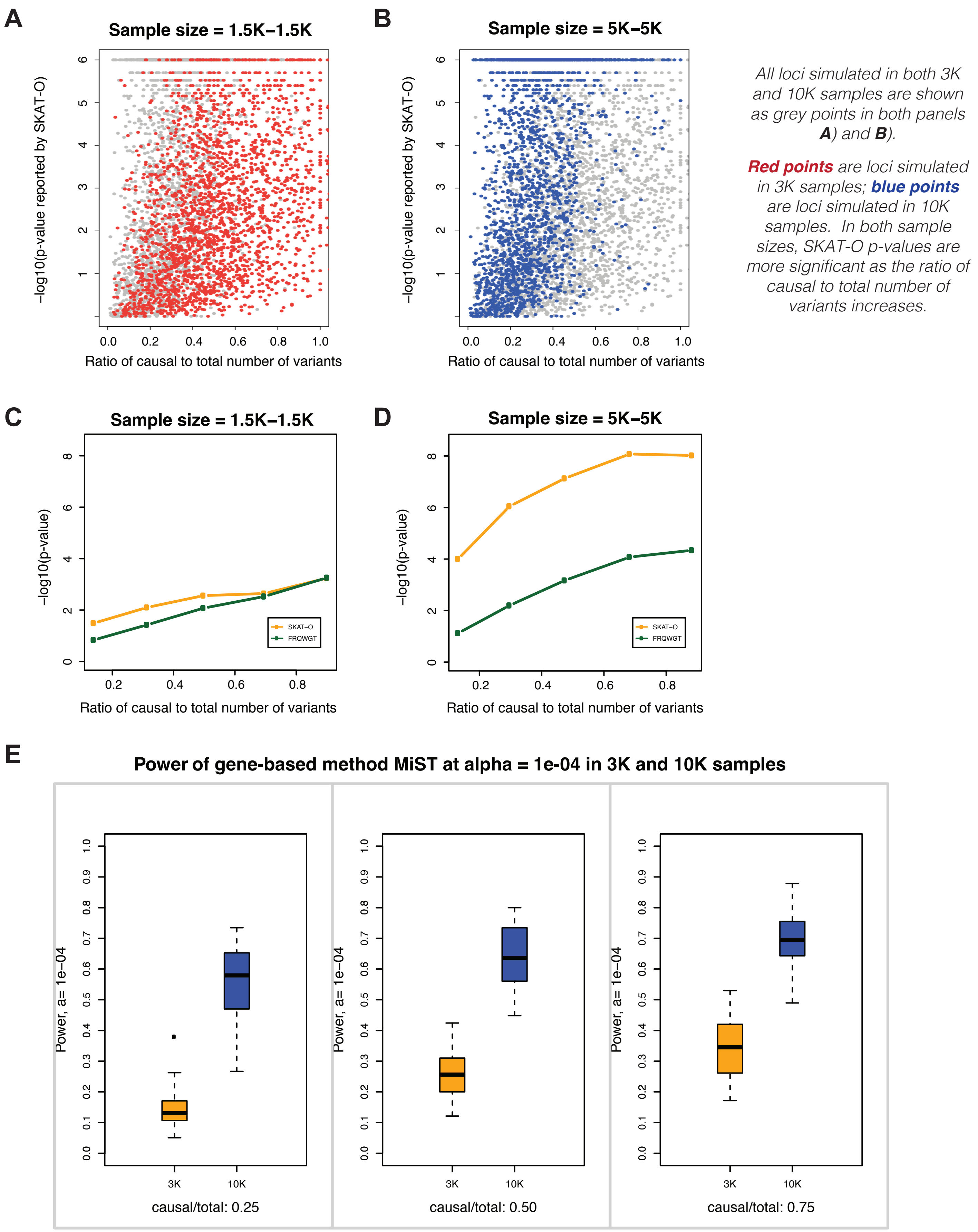

Supplement: S13 Fig — All results shown in this figure are for loci that explain 1% of phenotypic variance; gene-based tests run in 3K samples (1.5 cases / 1.5K controls) or in 10K samples (5K cases / 5K controls); loci simulated under AR2 (moderate selection). (A–B) Each point represents a simulated locus (across 24 human genes, 100 replicates per gene, simulations in both 3K and 10K samples; 4800 points are plotted in each scatterplot). The x-axis shows the ratio of number of causal variants (variants to which phenotypic effects are assigned) relative to total number of variants tested across the locus (all variants with MAF<1%). The y-axis shows p-values reported by the gene-based association method SKAT-O. All loci simulated in both 3K and 10K samples are shown as grey points in both panels (A) and (B). Red points are loci simulated in 3K samples; blue points are loci simulated in 10K samples. Blue points (loci simulated in 10K samples) are left shifted relative to red points (loci simulated in 3K samples) because the fraction of neutral variation increases with sample size in these simulations. Blue points are also shifted up, as p-values in 10K samples are more significant than those in 3K samples. In both sample sizes, SKAT-O p-values are more significant as the ratio of causal to total number of variants increases. (C–D) Mean p-values reported by two gene-based association methods (SKAT-O and FRQWGT) as a function of the proportion of causal variation at a locus, in 3K (C) and 10K (D) samples. In 3K samples, both methods show similar performance, but in 10K samples SKAT-O significantly outperforms FRQWGT method, regardless of the proportion of causal variation. While the lower mean power of FRQWGT in 10K samples may be partially attributable to the larger number of neutral variants in this sample size (S12 Fig), panel (D) suggests that FRQWGT may also be less-powered for other reasons (and is not entirely a result of bias introduced by the cap on the number of causal variants in [file pgen.1005165.s014.pdf]
