# Supplementary material for: The Power of Gene-Based Rare Variant Methods to Detect Disease-Associated Variation and Test Hypotheses About Complex Disease
Source: PLoS Genet. 2015 Apr 23;11(4):e1005165. doi: 10.1371/journal.pgen.1005165 (PMC4407972; doi:10.1371/journal.pgen.1005165)
Supplement: S14 Fig — All results shown here are for loci which explain 1% of phenotypic variance; gene-based tests run in 3K samples (1.5 cases / 1.5K controls). Values represent R2 correlation coefficients between p-values reported by each gene-based association method. Correlation coefficients are shown under all simulated architectures: (A) AR1, (B) AR2, (C) AR3, (D) AR4, (E) AR5, and (F) AR6. (PDF) [file pgen.1005165.s015.pdf]

S14 Figure: Concordance between p-values reported by different gene-based association methods under each simulated architecture.

A

AR1 (strong selection); VE per gene = 1%

	BURDEN	CALPHA	FRQWGT	KBAC	UNIQ	VT	skat	madsen	cmc	skat–o	MIST
BURDEN	1	0.28	0.76	0.87	0.1	0.81	0.28	0.96	0.96	0.74	0.64
CALPHA	0.28	1	0.05	0.43	0	0.15	0.99	0.23	0.26	0.69	0.65
FRQWGT	0.76	0.05	1	0.61	0.26	0.84	0.06	0.78	0.74	0.42	0.42
KBAC	0.87	0.43	0.61	1	0.1	0.71	0.44	0.87	0.88	0.81	0.77
UNIQ	0.1	0	0.26	0.1	1	0.2	0	0.1	0.09	0.04	0.06
VT	0.81	0.15	0.84	0.71	0.2	1	0.16	0.81	0.78	0.55	0.55
skat	0.28	0.99	0.06	0.44	0	0.16	1	0.24	0.26	0.7	0.67
madsen	0.96	0.23	0.78	0.87	0.1	0.81	0.24	1	1	0.69	0.61
cmc	0.96	0.26	0.74	0.88	0.09	0.78	0.26	1	1	0.71	0.62
skat–o	0.74	0.69	0.42	0.81	0.04	0.55	0.7	0.69	0.71	1	0.89
MIST	0.64	0.65	0.42	0.77	0.06	0.55	0.67	0.61	0.62	0.89	1

B

AR2 (moderate selection); VE per gene = 1%

	BURDEN	CALPHA	FRQWGT	KBAC	UNIQ	VT	skat	madsen	cmc	skat–o	MIST
BURDEN	1	0.41	0.8	0.89	0.15	0.84	0.41	0.96	0.95	0.8	0.71
CALPHA	0.41	1	0.16	0.56	0.02	0.29	0.99	0.36	0.39	0.77	0.75
FRQWGT	0.8	0.16	1	0.68	0.31	0.87	0.16	0.8	0.77	0.54	0.53
KBAC	0.89	0.56	0.68	1	0.16	0.77	0.58	0.88	0.89	0.86	0.84
UNIQ	0.15	0.02	0.31	0.16	1	0.26	0.02	0.15	0.13	0.09	0.13
VT	0.84	0.29	0.87	0.77	0.26	1	0.31	0.83	0.81	0.65	0.65
skat	0.41	0.99	0.16	0.58	0.02	0.31	1	0.37	0.39	0.78	0.77
madsen	0.96	0.36	0.8	0.88	0.15	0.83	0.37	1	1	0.76	0.69
cmc	0.95	0.39	0.77	0.89	0.13	0.81	0.39	1	1	0.77	0.7
skat–o	0.8	0.77	0.54	0.86	0.09	0.65	0.78	0.76	0.77	1	0.92
MIST	0.71	0.75	0.53	0.84	0.13	0.65	0.77	0.69	0.7	0.92	1

C

AR3 (weak selection); VE per gene = 1%

	BURDEN	CALPHA	FRQWGT	KBAC	UNIQ	VT	skat	madsen	cmc	skat–o	MIST
BURDEN	1	0.42	0.84	0.91	0.2	0.84	0.42	0.94	0.94	0.79	0.73
CALPHA	0.42	1	0.21	0.58	0.04	0.34	0.99	0.4	0.42	0.78	0.78
FRQWGT	0.84	0.21	1	0.74	0.34	0.88	0.21	0.81	0.79	0.59	0.58
KBAC	0.91	0.58	0.74	1	0.22	0.79	0.59	0.88	0.88	0.86	0.85
UNIQ	0.2	0.04	0.34	0.22	1	0.31	0.05	0.2	0.19	0.14	0.18
VT	0.84	0.34	0.88	0.79	0.31	1	0.35	0.82	0.8	0.67	0.68
skat	0.42	0.99	0.21	0.59	0.05	0.35	1	0.4	0.42	0.79	0.8
madsen	0.94	0.4	0.81	0.88	0.2	0.82	0.4	1	1	0.78	0.72
cmc	0.94	0.42	0.79	0.88	0.19	0.8	0.42	1	1	0.79	0.73
skat–o	0.79	0.78	0.59	0.86	0.14	0.67	0.79	0.78	0.79	1	0.94
MIST	0.73	0.78	0.58	0.85	0.18	0.68	0.8	0.72	0.73	0.94	1

D

AR4 (strong selection; only MAF<1% causal)

	BURDEN	CALPHA	FRQWGT	KBAC	UNIQ	VT	skat	madsen	cmc	skat–o	MIST
BURDEN	1	0.2	0.69	0.85	0.09	0.74	0.2	0.95	0.95	0.77	0.59
CALPHA	0.2	1	0.01	0.35	0.01	0.09	0.98	0.17	0.19	0.56	0.48
FRQWGT	0.69	0.01	1	0.56	0.27	0.82	0.01	0.72	0.68	0.39	0.42
KBAC	0.85	0.35	0.56	1	0.08	0.67	0.36	0.86	0.87	0.82	0.74
UNIQ	0.09	0.01	0.27	0.08	1	0.2	0.01	0.09	0.08	0.04	0.08
VT	0.74	0.09	0.82	0.67	0.2	1	0.1	0.75	0.71	0.53	0.59
skat	0.2	0.98	0.01	0.36	0.01	0.1	1	0.17	0.19	0.57	0.5
madsen	0.95	0.17	0.72	0.86	0.09	0.75	0.17	1	1	0.72	0.58
cmc	0.95	0.19	0.68	0.87	0.08	0.71	0.19	1	1	0.74	0.58
skat–o	0.77	0.56	0.39	0.82	0.04	0.53	0.57	0.72	0.74	1	0.81
MIST	0.59	0.48	0.42	0.74	0.08	0.59	0.5	0.58	0.58	0.81	1

E

AR5 (moderate selection; only MAF<1% causal)

	BURDEN	CALPHA	FRQWGT	KBAC	UNIQ	VT	skat	madsen	cmc	skat–o	MIST
BURDEN	1	0.26	0.72	0.86	0.09	0.82	0.26	0.96	0.96	0.81	0.65
CALPHA	0.26	1	0.03	0.41	0	0.15	0.98	0.23	0.25	0.58	0.52
FRQWGT	0.72	0.03	1	0.57	0.23	0.82	0.04	0.75	0.72	0.47	0.47
KBAC	0.86	0.41	0.57	1	0.08	0.72	0.42	0.86	0.87	0.85	0.78
UNIQ	0.09	0	0.23	0.08	1	0.16	0	0.09	0.08	0.05	0.08
VT	0.82	0.15	0.82	0.72	0.16	1	0.17	0.82	0.79	0.64	0.66
skat	0.26	0.98	0.04	0.42	0	0.17	1	0.23	0.25	0.59	0.54
madsen	0.96	0.23	0.75	0.86	0.09	0.82	0.23	1	1	0.77	0.64
cmc	0.96	0.25	0.72	0.87	0.08	0.79	0.25	1	1	0.78	0.64
skat–o	0.81	0.58	0.47	0.85	0.05	0.64	0.59	0.77	0.78	1	0.85
MIST	0.65	0.52	0.47	0.78	0.08	0.66	0.54	0.64	0.64	0.85	1

F

AR6 (moderate selection; bi-directional effects)

	BURDEN	CALPHA	FRQWGT	KBAC	UNIQ	VT	skat	madsen	cmc	skat–o	MIST
BURDEN	1	0.34	0.76	0.85	0.07	0.81	0.34	0.77	0.77	0.5	0.47
CALPHA	0.34	1	0.12	0.56	0.01	0.26	0.99	0.3	0.32	0.92	0.87
FRQWGT	0.76	0.12	1	0.61	0.22	0.8	0.12	0.58	0.56	0.25	0.27
KBAC	0.85	0.56	0.61	1	0.09	0.7	0.57	0.65	0.66	0.68	0.67
UNIQ	0.07	0.01	0.22	0.09	1	0.16	0.01	0.04	0.04	0.02	0.03
VT	0.81	0.26	0.8	0.7	0.16	1	0.26	0.64	0.63	0.4	0.41
skat	0.34	0.99	0.12	0.57	0.01	0.26	1	0.3	0.32	0.93	0.88
madsen	0.77	0.3	0.58	0.65	0.04	0.64	0.3	1	1	0.5	0.48
cmc	0.77	0.32	0.56	0.66	0.04	0.63	0.32	1	1	0.52	0.49
skat–o	0.5	0.92	0.25	0.68	0.02	0.4	0.93	0.5	0.52	1	0.93
MIST	0.47	0.87	0.27	0.67	0.03	0.41	0.88	0.48	0.49	0.93	1
