# Supplementary material for: The Power of Gene-Based Rare Variant Methods to Detect Disease-Associated Variation and Test Hypotheses About Complex Disease
Source: PLoS Genet. 2015 Apr 23;11(4):e1005165. doi: 10.1371/journal.pgen.1005165 (PMC4407972; doi:10.1371/journal.pgen.1005165)
Supplement: S2 Table — The below range of locus architectures were modeled at simulated loci; variant effect sizes were sampled from joint frequency-effect size distributions learned from forward population genetic simulations (Agarwala et al, Nature Genetics 2013). The architectures were chosen to reflect a range of different rare variant contributions and effect sizes. Briefly, the ‘selection parameter’ controls the degree to which the effect of a variant on evolutionary fitness is coupled to its effect on disease. When tau = 1, variants that are deleterious for fitness (and thus rarer in the population) have the largest effects on disease. When tau = 0, all causal variants have comparable additive effects on disease regardless of their effects on fitness, and thus common causal variants contribute a greater proportion of total heritability (S1 Text). At each locus, the total number of causal variants depended on the effect sizes sampled, as loci were modeled to explain a fixed proportion of liability-scale phenotypic variance underlying a complex trait with 8% prevalence (such as type 2 diabetes). (PDF) [file pgen.1005165.s017.pdf]

S2 Table: Locus architectures modeled at simulated loci.

Architecture	Direction of effects	Selection parameter (from Agarwala et al)	Description (also see S3 Figure)
AR1	All deleterious	tau = 1	Rare variants (MAF<1%) explain >90% of heritability and have large additive effects relative to common variants
AR2	All deleterious	tau = 0.5	Rare variants explain ~50% of heritability and have moderate additive effect sizes relative to common variants
AR3	All deleterious	tau = 0	Rare variants explain <15% of heritability and have additive effects comparable to common variants
AR4	All deleterious	tau = 1, all causal variants have MAF < 1%	Same effect size distribution as AR1; but only rare (MAF<1%) are causal at the simulated locus
AR5	All deleterious	tau = 0.5, all causal variants have MAF < 1%	Same effect size distribution as AR2; but only rare (MAF<1%) are causal at the simulated locus
AR6	50% deleterious, 50% protective	tau = 0.5	Same effect size distribution as AR2; but 50% of variants have protective effects on disease
