# Supplementary material for: The Power of Gene-Based Rare Variant Methods to Detect Disease-Associated Variation and Test Hypotheses About Complex Disease
Source: PLoS Genet. 2015 Apr 23;11(4):e1005165. doi: 10.1371/journal.pgen.1005165 (PMC4407972; doi:10.1371/journal.pgen.1005165)
Supplement: S3 Table — Test combinations were picked using step-wise forward selection starting from each of the three best-performing gene-based association methods across architectures (KBAC, SKAT-O, MiST). First column lists the architecture (see S2 Table and main text for more information). VE refers to total phenotypic variance explained by the locus; D/M describes whether causal effects are deleterious only (D) or a mix of deleterious and protective (M). The second column contains the ‘starting’ test, and the third column indicates the ‘margin’ of difference in p-values used in the forward selection algorithm (where 100 is 2 orders of magnitude, 10 is 1 order of magnitude and 1 is minimum margin; see S1 Text). The fourth column contains the list of tests picked by the selection algorithm until no other addition offered higher power under that margin, in the order of selection. Column 5 shows the total sensitivity of the composite test (when the minimum p-value across all tests in the group is used), using an (un-adjusted) p-value threshold of 1e-04. Column 6 shows power under the adjusted p-value threshold (to maintain a false positive rate < = 1e-04). Column 7 shows power of the single best gene-based test for comparison (at alpha = 1e-04). For each architecture, the best-performing ‘composite’ test is shown in bold. (PDF) [file pgen.1005165.s018.pdf]

S3 Table: Power of 'composite' groups of gene-based association methods.

AR, VE%, D/M	Starting test	Margin	Tests (in order of inclusion)	Composite Sensitivity	Power (adj. p-value)	Mean power of single best test
AR1, 1%, D	KBAC	100	KBAC,CALPHA,UNIQ.VT	0.431	0.348	0.359 (MiST)
AR1, 1%, D	KBAC	10	KBAC,MiST,VT,UNIQ,CALPHA,FRQWGT	0.465	0.360	0.359
AR1, 1%, D	KBAC	1	KBAC,MiST,VT,FRQWGT,CALPHA,UNIQ,BURDEN,SKATO	0.468	0.365	0.359
AR1, 1%, D	SKATO	100	SKATO, VT,UNIQ,MiST	0.420	0.356	0.359
**AR1, 1%, D**	**SKATO**	**10**	**SKATO,VT,UNIQ,MiST,KBAC,FRQWGT**	**0.459**	**0.373**	**0.359**
AR1, 1%, D	SKATO	1	SKATO,KBAC,MiST,VT,FRQWGT,UNIQ,CALPHA,BURDEN	0.468	0.365	0.359
AR1, 1%, D	MiST	100	MiST,CALPHA,UNIQ,VT	0.423	0.346	0.359
AR1, 1%, D	MiST	10	MiST,VT,UNIQ,CALPHA,KBAC,FRQWGT	0.465	0.360	0.359
AR1, 1%, D	MiST	1	MiST,KBAC,VT,FRQWGT,CALPHA,UNIQ,BURDEN,SKATO	0.468	0.365	0.359
AR2, 1%, D	KBAC	100	KBAC,CALPHA,UNIQ,FRQWGT	0.297	0.229	0.231 (MiST)
AR2, 1%, D	KBAC	10	KBAC,CALPHA,UNIQ,FRQWGT,MiST	0.315	0.245	0.231
AR2, 1%, D	KBAC	1	KBAC,MiST,FRQWGT,CALPHA,UNIQ.BURDEN,VT,SKATO,WILCOX-WSS	0.327	0.243	0.231
AR2, 1%, D	SKATO	100	SKATO,UNIQ,MiST	0.258	0.245	0.231
**AR2, 1%, D**	**SKATO**	**10**	**SKATO,VT,KBAC,UNIQ,FRQWGT,MiST**	**0.310**	**0.245**	**0.231**
AR2, 1%, D	SKATO	1	SKATO,KBAC,MiST,FRQWGT,CALPHA,UNIQ,BURDEN,VT,WILCOX-WSS	0.327	0.243	0.231
AR2, 1%, D	MiST	100	MiST,CALPHA,UNIQ	0.257	0.208	0.231
AR2, 1%, D	MiST	10	MiST,VT,KBAC,UNIQ,FRQWGT	0.308	0.244	0.231
AR2, 1%, D	MiST	1	MiST,KBAC,FRQWGT,CALPHA,UNIQ,BURDEN,VT,SKATO,WILCOX-WSS	0.327	0.243	0.231
AR3, 1%, D	KBAC	100	KBAC, UNIQ, CALPHA	0.120	0.091	0.095 (MiST)
AR3, 1%, D	KBAC	10	KBAC,CALPHA,UNIQ,VT, WILCOX-WSS	0.128	0.097	0.095
AR3, 1%, D	KBAC	1	KBAC,MiST,FRQWGT,UNIQ,VT,CALPHA,BURDEN,SKAT,WILCOX-WSS,CMC	0.142	0.103	0.095
AR3, 1%, D	SKATO	100	SKATO,VT,UNIQ,CALPHA	0.115	0.090	0.095
AR3, 1%, D	SKATO	10	SKATO,UNIQ,VT,CALPHA,KBAC	0.130	0.097	0.095
AR3, 1%, D	SKATO	1	SKATO,KBAC,MiST,FRQWGT,UNIQ,VT,CALPHA,BURDEN,WILCOX-WSS,CMC	0.143	0.103	0.095
**AR3, 1%, D**	**MiST**	**100**	**MiST,UNIQ,BURDEN**	**0.123**	**0.110**	**0.095**
AR3, 1%, D	MiST	10	MiST,UNIQ,BURDEN,VT	0.126	0.102	0.095
AR3, 1%, D	MiST	1	MiST,KBAC,FRQWGT,UNIQ,VT,CALPHA,BURDEN,SKAT,WILCOX-WSS,CMC	0.142	0.103	0.095
AR5, 1%, D	KBAC	100	KBAC,UNIQ,CALPHA,VT	0.593	0.484	0.500 (MiST)
AR5, 1%, D	KBAC	10	KBAC,MiST,UNIQ,VT,FRQWGT,CALPHA	0.635	0.499	0.5
AR5, 1%, D	KBAC	1	KBAC,MiST,VT,UNIQ,BURDEN,FRQWGT,CALPHA,SKATO	0.639	0.508	0.5
AR5, 1%, D	SKATO	100	SKATO,UNIQ,VT	0.550	0.470	0.5
**AR5, 1%, D**	**SKATO**	**10**	**SKATO,VT,UNIQ,KBAC,MiST,FRQWGT**	**0.630**	**0.520**	**0.5**
AR5, 1%, D	SKATO	1	SKATO,VT,MiST,KBAC,UNIQ,BURDEN,FRQWGT,CALPHA	0.639	0.508	0.5
AR5, 1%, D	MiST	100	MiST,CALPHA,UNIQ,VT	0.586	0.492	0.5
AR5, 1%, D	MiST	10	MiST,UNIQ,KBAC,VT,FRQWGT,CALPHA	0.635	0.499	0.5
AR5, 1%, D	MiST	1	MiST,VT,KBAC,UNIQ,BURDEN,FRQWGT,CALPHA,SKATO	0.639	0.508	0.5
AR6, 1%, D	KBAC	100	KBAC,CALPHA,UNIQ	0.546	0.465	0.498 (MiST)
AR6, 1%, D	KBAC	10	KBAC,MiST,UNIQ,FRQWGT,CALPHA,VT	0.627	0.510	0.498
AR6, 1%, D	KBAC	1	KBAC,MiST,FRQWGT,VT,UNIQ,CALPHA,BURDEN,SKATO	0.635	0.515	0.498
AR6, 1%, D	SKATO	100	SKATO,UNIQ,VT	0.530	0.455	0.498
**AR6, 1%, D**	**SKATO**	**10**	**SKATO,VT,UNIQ,KBAC,MiST**	**0.615**	**0.518**	**0.498**
AR6, 1%, D	SKATO	1	SKATO,KBAC,VT,MiST,FRQWGT,UNIQ,CALPHA,BURDEN	0.635	0.515	0.498
AR6, 1%, D	MiST	100	MiST,CALPHA,UNIQ	0.525	0.451	0.498
AR6, 1%, D	MiST	10	MiST,VT,UNIQ,KBAC,CALPHA	0.615	0.516	0.498
AR6, 1%, D	MiST	1	MiST,KBAC,FRQWGT,VT,UNIQ,CALPHA,BURDEN,SKATO	0.635	0.515	0.498
AR2, 1%, M	KBAC	100	KBAC,CALPHA,UNIQ	0.113	0.088	0.093 (CALPHA)
AR2, 1%, M	KBAC	10	KBAC,CALPHA,UNIQ,MiST	0.118	0.094	0.093
AR2, 1%, M	KBAC	1	KBAC,CALPHA,MiST,UNIQ,BURDEN,VT,SKAT	0.121	0.089	0.093
**AR2, 1%, M**	**SKATO**	**100**	**SKATO,UNIQ,MiST**	**0.097**	**0.094**	**0.093 (CALPHA)**
AR2, 1%, M	SKATO	10	SKATO,UNIQ,MiST,FRQWGT	0.098	0.083	0.093
AR2, 1%, M	SKATO	1	SKATO,CALPHA,KBAC,MiST,UNIQ,BURDEN,VT,SKAT	0.121	0.089	0.093
AR2, 1%, M	MiST	100	MiST,CALPHA,UNIQ	0.109	0.086	0.093
AR2, 1%, M	MiST	10	MiST,UNIQ,KBAC,CALPHA	0.118	0.094	0.093
AR2, 1%, M	MiST	1	MiST,CALPHA,KBAC,UNIQ,BURDEN,VT,SKAT	0.121	0.089	0.093
AR2, 0.5%, D	KBAC	100	KBAC,UNIQ,CALPHA,VT	0.065	0.040	0.041 (MiST)
AR2, 0.5%, D	KBAC	10	KBAC,CALPHA,UNIQ,VT,MiST	0.071	0.042	0.041
AR2, 0.5%, D	KBAC	1	KBAC,MiST,CALPHA,VT,BURDEN,UNIQ,FRQWGT,SKATO	0.075	0.039	0.041
AR2, 0.5%, D	SKATO	100	SKATO,UNIQ,VT	0.050	0.034	0.041
AR2, 0.5%, D	SKATO	10	SKATO,VT,UNIQ,MiST,KBAC	0.065	0.040	0.041
AR2, 0.5%, D	SKATO	1	SKATO,MiST,KBAC,VT,CALPHA,BURDEN,UNIQ,FRQWGT	0.075	0.039	0.041
**AR2, 0.5%, D**	**MiST**	**100**	**MiST,UNIQ,BURDEN**	**0.057**	**0.047**	**0.041**
AR2, 0.5%, D	MiST	10	MiST,UNIQ,KBAC,VT	0.064	0.038	0.041
AR2, 0.5%, D	MiST	1	MiST,KBAC,CALPHA,VT,BURDEN,UNIQ,FRQWGT,SKATO	0.075	0.039	0.041
